# Theoretical Studies on the Engagement of Interleukin 18 in the Immuno-Inflammatory Processes Underlying Atherosclerosis

**DOI:** 10.3390/ijms19113476

**Published:** 2018-11-05

**Authors:** Dorota Formanowicz, Kaja Gutowska, Piotr Formanowicz

**Affiliations:** 1 Department of Clinical Biochemistry and Laboratory Medicine, Poznan University of Medical Sciences, Rokietnicka 8, 60-806 Poznan, Poland; doforman@ump.edu.pl; 2 Institute of Computing Science, Poznan University of Technology, Piotrowo 2, 60-965 Poznan, Poland; Kaja.Gutowska@cs.put.poznan.pl; 3 Institute of Bioorganic Chemistry, Polish Academy of Sciences, Noskowskiego 12/14, 61-704 Poznan, Poland

**Keywords:** interleukin 18, macrophages, atherosclerosis, modeling, Petri nets, t-invariants

## Abstract

Interleukin 18 (IL-18) is one of the pro-inflammatory cytokines expressed by macrophages, suggesting that it plays important physiological and immunological functions, among the others: stimulation of natural killers (NKs) and T cells to interferon gamma (IFN-γ) synthesis. IL-18 was originally identified as interferon gamma inducing factor and now it is recognized as multifunctional cytokine, which has a role in regulation of innate and adaptive immune responses. Therefore, in order to investigate IL-18 contribution to the immuno-inflammatory processes underlying atherosclerosis, a systems approach has been used in our studies. For this purpose, a model of the studied phenomenon, including selected pathways, based on the Petri-net theory, has been created and then analyzed. Two pathways of IL-18 synthesis have been distinguished: caspase 1-dependent pathway and caspase 1-independent pathway. The analysis based on t-invariants allowed for determining interesting dependencies between IL-18 and different types of macrophages: M1 are involved in positive regulation of IL-18, while M2 are involved in negative regulation of IL-18. Moreover, the obtained results showed that IL-18 is produced more often via caspase 1-independent pathway than caspase 1-dependent pathway. Furthermore, we found that this last pathway may be associated with caspase 8 action.

## 1. Introduction

Atherosclerosis, a chronic inflammatory disease triggered by lipid retention in the wall of the arteries, resulting from the complex interactions of many biological pathways, is one of the most widespread diseases in the world. The crosstalk between inflammatory cells of atherosclerotic plaques results in the production of high levels of inflammatory cytokines that are responsible for the detrimental effect on the composition of atherosclerotic plaques. One of them, responsible for destabilization of atherosclerotic plaque, is interleukin-18 (IL-18).

This cytokine is a unique one. Our interest in the IL-18 engagement in the immuno-inflammatory processes underlying atherosclerosis is based on several important findings. Firstly, IL-18 is one of the most important innate cytokines. It was revealed that it can initiate cascade of pro-inflammatory cytokines through the production of immediate early inflammatory cytokines such as tumor necrosis factor α (TNF-α) and interleukin 1β (IL-1β). IL-18 was originally identified as interferon gamma (IFN-γ) inducing factor, which was found to support atherosclerotic plaque development and its instability [1]. Namely, IFN-γ contributes to the thinning of the fibrous cap of atherosclerotic plaque, due to the stimulation of the expression of adhesion molecules on endothelial cells and major histocompatibility complex II (MHC II) on macrophages and vascular cells as well as the inhibition of collagen synthesis, which upregulates the expression of matrix metalloproteinases [2]. However, it should be underlined that IL-18 not only promotes IFN-γ synthesis, but also likely participates in its overall activities. It induces Th1-mediated immune responses in co-stimulation with other Th1-related cytokines [3]. Alternatively, in synergy with IL-23, it activates Th17 cells to produce IL-17, cytokine which recruits monocytes and neutrophils to the site of inflammation [4]. In the absence of known synergies, IL-18 can activate Th2 response by IL-4 and IL-13 production [5].

Since the adaptive immune system is involved in the development of atherosclerosis, deficiency in both T and B cells significantly inhibits atherosclerotic lesions development. The majority of pathogenic T cells in atherosclerosis are of the Th1 profile producing high levels of IFN-γ. Hence, induction of IFN-γ may explain the critical role of IL-18 in atherosclerosis. On the other hand, the available data suggest that potentially Th2-mediated responses also contribute to the development and progression of atherosclerosis. Therefore, identification of the causes of Th1-Th2 dysregulation would be of great importance to a better understanding of the pathophysiology of atherosclerosis. IL-18 stimulates Th1 or Th2 responses depending on its cytokine milieu [4], so its changes may be crucial for the atherosclerotic process.

In this study, we have focused on the dependencies between IL-18, cytokine responsible for the harmful effect on the composition of atherosclerotic lesions [6], and macrophages characterized by high heterogeneity and versatility of the immune response. The overall macrophage functionality is critical for the balance between atherosclerotic plaque progression and regression [7]. They can polarize into different functional phenotypes in response to various environmental cues (e.g., microbial products, damaged cells, activated lymphocytes) or in various pathological conditions. Therefore, macrophages can differentiate into the opposing activities of killing or repairing, either a pro-inflammatory M1-like subtype, also known as a classically activated, or an anti-inflammatory alternatively activated subtype, M2-like macrophages [8].

The methodology of classical experimental medicine is based mainly on the study of single factors and the treatment of organisms as simple and linear systems. This solution, in case of study of interaction network between selected inflammatory cells within the endothelium, does not seem to be appropriate, thus we applied a systems approach. One of the important assumptions of the systems approach is to create the most reliable models that cover key processes occurring in the organism [9]. For this purpose, a mathematical model based on Petri net theory has been created [10,11,12]. The analyses of the model was based on t-invariants (corresponding to subprocesses that occur in the modeled phenomenon) and Maximal Common Transition (MCT) sets (see the Methods section).

Searching for the similarities between t-invariants allowed for determining that IL-18 is more often produced via the caspase 1-independent pathway than the caspase 1-dependent pathway. Moreover, we have found that the caspase 1-independent pathway may be associated with caspase 8. Furthermore, the obtained results showed interesting dependencies between different types of macrophages and the studied cytokine.

## 2. Analysis

Concepts and definitions concerning Petri nets which are necessary for understanding the presented analyses are described in Methods section. All descriptions of the elements of the presented model, both places and transitions, are explained in Table A1 and Table A2 in appendices. All the abbreviations used are explained in these tables.

### 2.1. Analysis of MCT Sets

The model includes 83 places, 97 transitions and 22 non-trivial Maximal Common Transition (MCT) sets (i.e., the ones containing more than one transition). For each MCT set, a biological interpretation is presented in Table 1.

### 2.2. Analysis of t-Clusters

The proposed Petri net based model is covered by 78 t-invariants, which are the basis of the analysis. Mean Split Silhouette index (MSS) and Calinski–Harabasz (C–H) coefficient has been used [13]. MSS evaluates a fit of each t-invariant to its cluster and an average quality of a given clustering [14]. The C–H coefficient has been calculated in the range from 2 to 20 clusters and the optimal number of clusters is indicated by the highest value [15]. For the biological analysis, 15 t-clusters have been chosen using an average linkage method and the Pearson measure of distance. t-clusters correspond to biological mechanisms and therefore an interpretation for each cluster is assigned in Table 2. Additionally, the appendix includes Table A3 presenting a range of t-invariants that represents each t-cluster. t-invariants have been sorted according to the similarity measure.

### 2.3. More Detailed Analysis of Particular t-Invariants

#### 2.3.1. Interleukin 18

**Figure 1:**
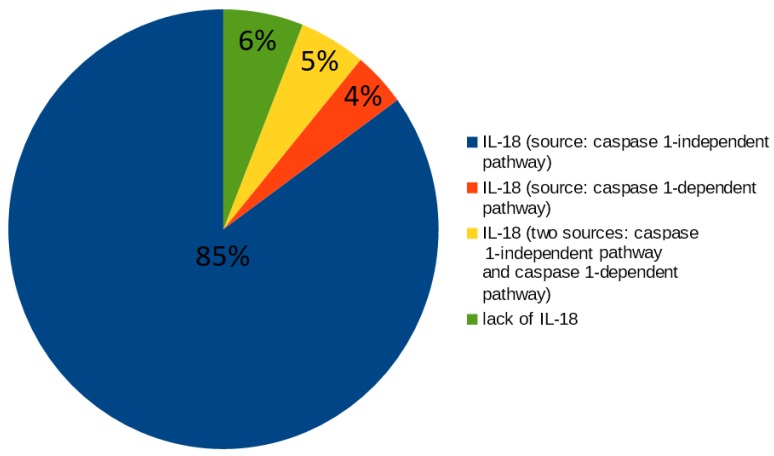
The diagram presents a count of t-invariants corresponding to subprocesses in which IL-18 produced by different pathways is engaged.

The analysis of t-clusters for the proposed model revealed that 12 (80%) out of 15 clusters include t-invariants corresponding to the subprocesses in which IL-18 is involved. The more detailed analysis is a manual method, which is based on searching for particular t-invariants in every t-cluster. This method leads to determination, which, of the subprocesses, are crucial for the modeled system, which is confirmed by the high percentage value. In this case, the more detailed analysis of t-invariants showed that 94% of t-invariants correspond to subprocesses in which IL-18 is engaged. To be more precise, IL-18 is associated with almost all mechanisms and processes, which are modeled in the proposed Petri net based model (the results are presented in Figure 1):

- 66 t-invariants (85%) correspond to subprocesses in which IL-18 produced by caspase 1-independent pathway is involved;
- 3 t-invariants (4%) correspond to subprocesses in which IL-18 produced by caspase 1-dependent pathway is involved;
- 4 t-invariants (5%) correspond to subprocesses in which IL-18 is produced by the two mentioned pathways (caspase 1-independent pathway and caspase 1-dependent pathway);
- 5 t-invariants (6%) correspond to subprocesses in which IL-18 is not involved.

**Figure 2:**
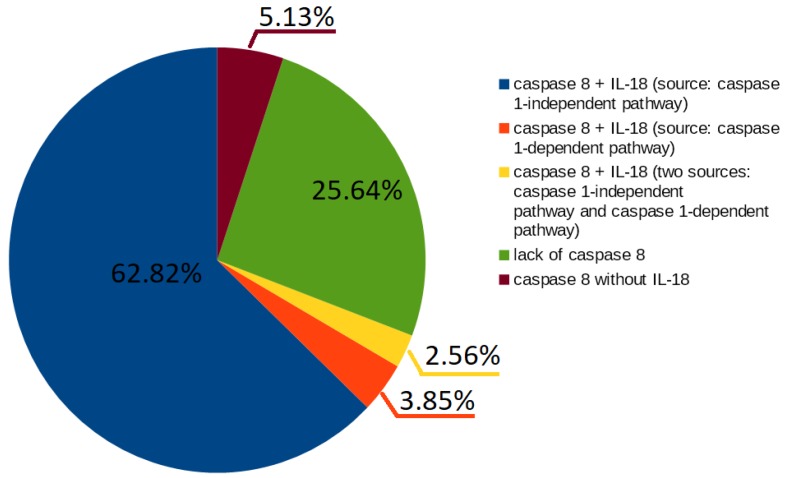
The diagram presents a count of t-invariants corresponding to subprocesses in which caspase 8 is engaged.

Caspase 8 presence is also interesting; 74% t-invariants correspond to subprocesses in which caspase 8 is engaged. Furthermore, caspase 1-independent pathway seems to be associated with caspase 8. More than half of t-invariants (62.82%) correspond to subprocesses in which caspase 8 occurs along with IL-18 produced by caspase-1 independent pathway (these results have been presented in Figure 2):

- 49 t-invariants (62.82%) correspond to subprocesses in which caspase 8 occurs along with IL-18 produced by caspase 1-independent pathway;
- 3 t-invariants (3.85%) correspond to subprocesses in which caspase 8 occurs along with IL-18 produced by caspase 1-dependent pathway;
- 2 t-invariants (2.56%) correspond to subprocesses in which caspase 8 occurs along with IL-18 produced by the two mentioned pathways (caspase 1-independent pathway and caspase 1-dependent pathway);
- 20 t-invariants (25.64%) correspond to subprocesses in which caspase 8 is not involved;
- 4 t-invariants (5.13%) correspond to subprocesses in which only caspase 8 is involved.

#### 2.3.2. Macrophages M1 and M2

**Figure 3:**
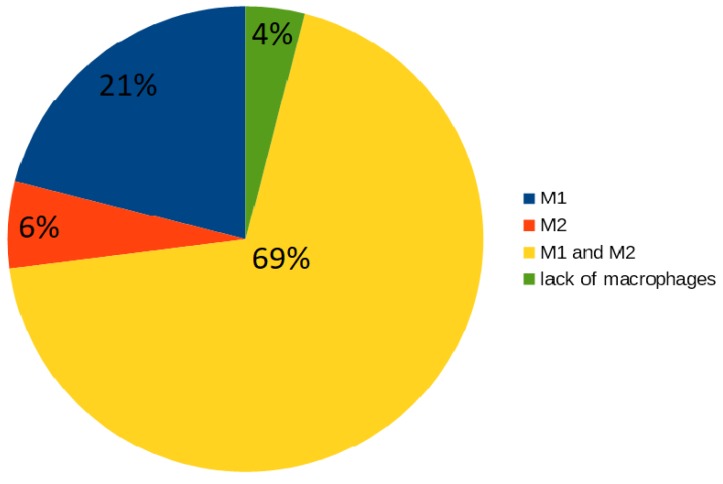
The diagram presents a count of t-invariants corresponding to subprocesses in which different types of macrophages are engaged.

Thirteen (87%) out of 15 t-clusters include t-invariants corresponding to subprocesses in which macrophages are engaged: one cluster includes type M1 of macrophages, four clusters include type M2 and eight clusters include both types of macrophages. The analysis of t-invariants showed that 96% t-invariants correspond to subprocesses in which macrophages are engaged. To be precise, macrophages are associated with almost all mechanisms, which are included in the proposed model (these results have been presented in Figure 3):

- 16 t-invariants (21%) correspond to subprocesses in which type M1 is involved;
- 5 t-invariants (6%) correspond to subprocesses in which type M2 is involved;
- 54 t-invariants (69%) correspond to subprocesses in which two types of macrophages are involved;
- 3 t-invariants (4%) correspond to subprocesses in which macrophages are not involved.

A more detailed analysis of t-invariants allows to determine subprocesses in which macrophages are involved:

- macrophages M1:
  - inhibition of caspase 8,
  - positive regulation of IL-18 (NO synthesis inhibition),
  - pathway of IκB phosphorylation by complex of receptors MyD88;
- macrophages M2:
  - activation of caspase 3, 6, 7 and 8,
  - high level of interleukin 10,
  - negative regulation of interleukin 18 (inhibition of IL-18),
  - apoptosis enhancement and anti-inflammation response.

#### 2.3.3. Knockout Analysis

The knockout analysis based on t-invariants has been performed in the context of various types of macrophages and it was possible using free (licensed under the Artistic License 2.0), stand-alone Java application Holmes [16]. Such an analysis allows for answering the question: which subprocesses will be excluded from the modeled system, if, for some reasons, the selected transition (some reaction) will be deactivated. Selected articles that used knockout analysis are [17,18]. The knockout analysis concerned two different types of macrophages: M1 and M2. Induction of activation of both M1 and M2 macrophages is possible through various independent mechanisms and reactions corresponding to the following transitions: $t_{33}$, $t_{74}$, $t_{95}$ for macrophages M1 and $t_{61}$, $t_{84}$ for macrophages M2.

The obtained results showed that the key transition enhancing induction of activation of macrophages M1 is transition $t_{33}$. Deactivation of this transition results in excluding 87% of subprocesses (68 out of 78 t-invariants), while deactivation of transition $t_{74}$ turns off 60% (47 t-invariants), and $t_{95}$ turns off about 9% (seven t-invariants) of all subprocesses. Subsequently, all three mentioned transitions leading to activation of M1 macrophages were simultaneous deactivated and as a result 70 of 78 subprocesses were excluded, i.e., about 90% of them. The more detailed analysis for the remaining t-invariants has been performed because five t-invariants out of eight correspond to subprocesses in which IL-18 is involved, so further analysis has been focused on them only. In five t-invariants corresponding to the IL-18 regulation, with deactivation of transitions corresponding to M1 macrophages, the majority is related to the negative regulation of IL-18, more precisely:

- 4 t-invariants (80%) correspond to subprocesses in which IL-18 is negatively regulated;
- 1 t-invariant (20%) corresponds to a subprocess in which IL-18 is positively regulated.

In the case of alternatively activated macrophages M2, both transitions exclude a similar number of subprocesses. Deactivation of transition $t_{61}$ disables about 33% of all subprocesses (26 out of 78 t-invariants), while deactivation of transition $t_{84}$ excludes about 37% of subprocesses (29 out of 78 t-invariants). The simultaneous deactivation of both transitions leads to the exclusion of 63% of all modeled subprocesses (49 out of 78 t-invariants). The more detailed analysis for the remaining t-invariants has been performed because 28 t-invariants out of 29 t-invariants correspond to subprocesses in which IL-18 is involved, so further analysis has been focused on them only. In 28 t-invariants corresponding to the IL-18 regulation, with deactivation of transitions corresponding to M2 macrophages, the majority is related to the positive regulation of IL-18, more precisely:

- 22 t-invariants (79%) correspond to subprocesses in which IL-18 is positively regulated;
- 6 t-invariants (21%) correspond to subprocesses in which IL-18 is negatively regulated.

The results of the knockout analysis are consistent with the results that have been presented in Section 2.3.2. When transitions corresponding to the activation of macrophages M1 have been excluded, most of the remaining subprocesses were associated with negative regulation of IL-18. When transitions corresponding to the activation of macrophages M2 have been excluded, most of the remaining subprocesses were associated with positive regulation of IL-18. The obtained results suggest that macrophages M1 have an influence on positive regulation of IL-18, whereas macrophages M2 have an influence on negative regulation of IL-18.

#### 2.3.4. Comparison of t-Clusters Analysis and the More Detailed Analysis of Particular t-Invariants

Five (33%) out of 15 t-clusters include t-invariants corresponding to almost all modeled mechanisms (Table 2). To obtain additional information, a more detailed analysis of particular t-invariants has been performed. This analysis has been focused on IL-18 and an influence of macrophages on the modeled phenomena. Comparison of these analyses (t-clusters analysis and the more detailed analysis of particular t-invariants) are provided in Table 3. This table presents the number of t-clusters and t-invariants that correspond to the subprocesses in which IL-18 and macrophages are involved. The results presented in Table 3 confirm that t-clusters analysis of the modeled biological process may be too general. However, these results also show that IL-18 and macrophages are engaged in almost all mechanisms that occur in the modeled system:

- 73 (94%) out of 78 t-invariants correspond to subprocesses in which IL-18 is produced;
- 75 (96%) out of 78 t-invariants correspond to subprocesses in which macrophages M1 and M2 are engaged.

## 3. The Model of Phenomena That Underlie the Formation of Atherosclerotic Lesions in the Arterial Subendothelial Region

**Figure 4:**
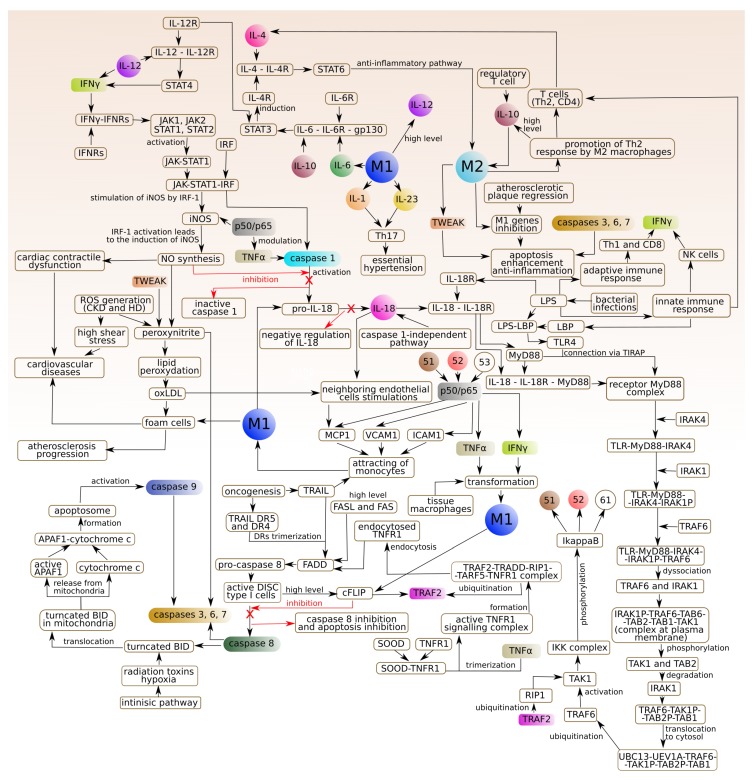
The diagram of the phenomena underlying the atherosclerotic lesions formation. The elements which are marked with the same colour and name correspond to the same particle (logic place). Element “51” corresponds to p50/p65 proteasome inhibitors induce inhibitory κB (IκB) phosphorylated complex within macrophages. Element “52” corresponds to p50/p65 dimer nuclear factor kappa-light-chain-enhancer of activated B cells (NF-κB) early phase in activated endothelial cell (EC) in atherosclerosis. Element “53” corresponds to p50/p65 dimer nuclear factor kappa-light-chain-enhancer of activated B cells (NF-κB)) early phase in activated smooth muscle cells (SMC). Element “61” corresponds to p50/p65 proteasome inhibitors induce inhibitory κB (IκB) phosphorylated complex in activated SMC. Element “p50/p65” corresponds to p50/p60 translocation to the nucleus in macrophages M1 and SMC and endothelial cells (EC).

To better understand the intricacies of the studied phenomenon and its biological background, the process is shown in the diagram (see Figure 4).

**Figure 5:**
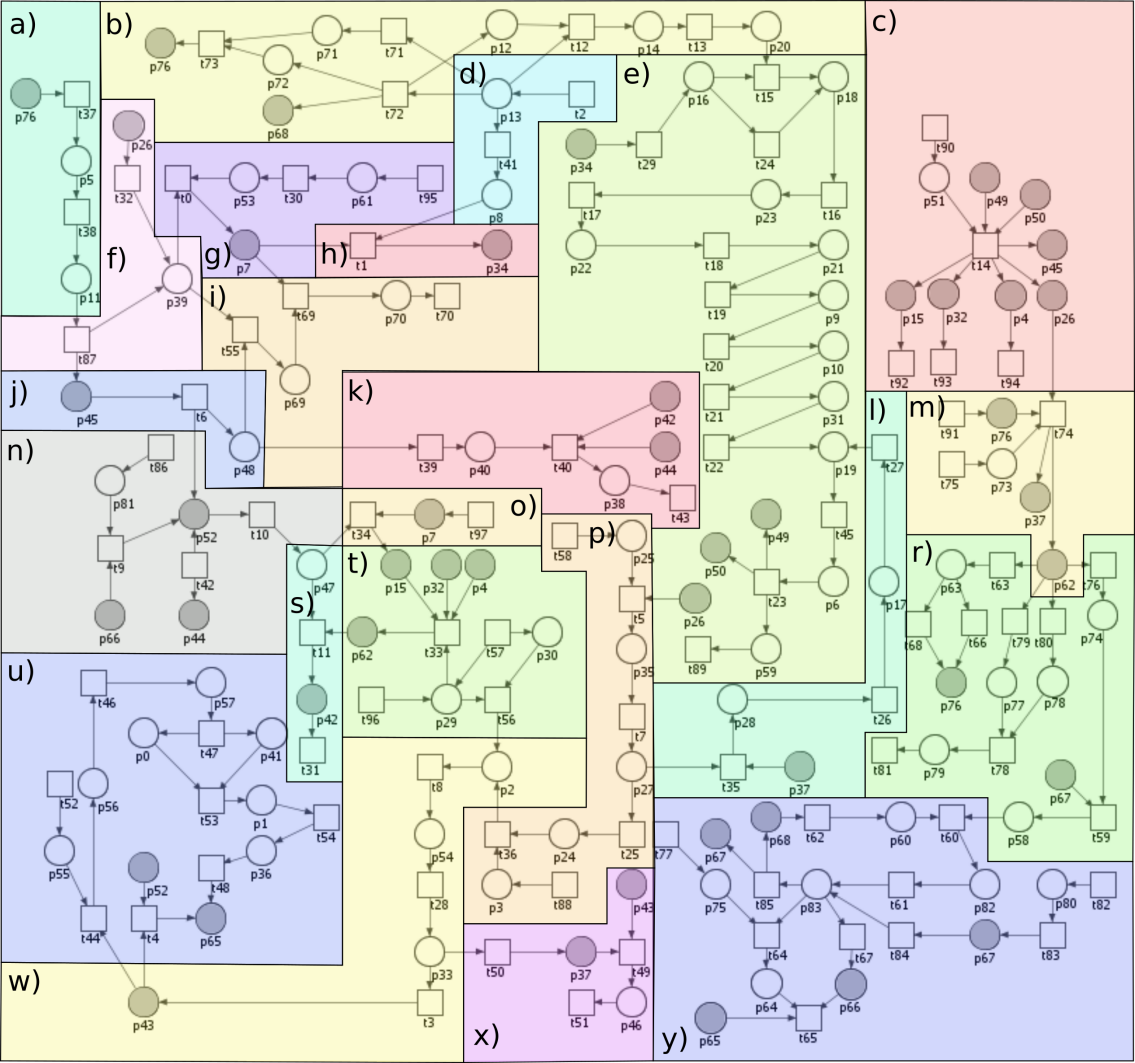
The proposed model has been divided into parts corresponding to the biological phenomena: (**a**) Janus kinases and signal transducer and activator of transcription (JAK-STAT) pathway stimulated by interferon gamma (IFNγ); (**b**) innate immune responses; (**c**) p50/p65 translocation to the nucleus in macrophages M1, smooth muscle cell (SMC) and endothelial cell (EC); (**d**) gram-negative bacterial infections; (**e**) activation of nuclear factor kappa-light-chain-enhancer of activated B cells (NF-κB)—the canonical pathway via IκBα phosphorylation by kinase complex (IKK) (MyD88-dependent signaling pathway); (**f**) activation of caspase 1 (by tumor necrosis factor (TNF) and also by binding with STAT binding element (SBE) sequences of interferon regulatory factor I (IRF1); (**g**) IL-18 synthesis (caspase 1-dependent pathway); (**h**) IL-18-IL-18R complex formation; (**i**) negative regulation of IL-18 (by inhibition of caspase 1) caused by nitric oxide (NO); (**j**) NO synthesis; (**k**) cardiovascular disease influenced by NO-dependent pathway; (**l**) TNF receptor-associated factor 2 (TRAF2) and receptor-interacting serine/threonine-protein (kinase 1 (RIP1) ubiquitination; (**m**) macrophages polarization; (**n**) lipid peroxidation (oxLDL); (**o**) IL-18 synthesis (caspase 1-independent pathway) and neighboring endothelial cells stimulation; (**p**) regulation of TNFR1 signaling; (**r**) the role of IL-1, IL-23, IL-6, high level of IL-10 and IL-12 (released by classically activated macrophages M1); (**s**) atherosclerosis progression; (**t**) attracting of monocytes (classically activated macrophages M1); (**u**) formation of apoptosome and activation of effector caspases 3, 6, 7; (**w**) high level of active caspase 8; (**x**) inhibition of caspase 8 and inhibition of apoptosis (by a high level of cFLIPs); (**y**) STAT6 upregulation promotes M2 macrophage polarization to suppress atherosclerosis.

The proposed model, based on the Petri net theory, created using the Snoopy software (1.21, Brandenburg University of Technology Cottbus (Data Structures and Software Dependability Group), Germany) [19], has been presented in Figure 5. To improve the readability of the model, it has been divided into 23 parts that correspond to the biological modules described below. These modules should be regarded as coupled blocks creating a full complex model of interactions.

Module (a) JAK-STAT pathway stimulated by interferon gamma (IFNγ):

IFNγ triggers antiviral and adaptive immune responses through the JAK-STAT signaling pathway, a highly conserved intracellular signaling pathway, which provides a simple and a direct route from the membrane receptors to the nucleus for mediating cellular responses to multiple cytokines [20,21,22].

Modules (b) and (d) Innate immune responses and Gram-negative bacterial infections:

Innate immunity provides a first line of defense against microbial invaders and relies on a family of pattern recognition receptors (PRRs). Some of the unique cell-wall components of bacteria stimulate immune cells and serve as pathogen-associated molecular patterns, recognized by individual Toll-like receptors (TLRs). Lipopolysaccharide (LPS) is responsible for most of the pathogenic phenomena associated with Gram-negative bacterial infection. It is released from Gram-negative bacteria and binds to the LPS binding protein (LBP), an acute phase protein present in the bloodstream, and then binds to CD14, a protein expressed on the surface of the phagocytes. LPS is then transferred to MyD2 that binds to the extracellular portion of TLR4 followed by oligomerization of TLR4. This results in initiation of the immune response via signal through adaptor proteins containing a Toll/interleukin-1 (TIR) domain [23]. Myeloid differentiation primary response gene 88 (MyD88) adaptor-like (MAL)/TIR domain-containing adaptor protein (TIRAP) is involved in bridging MyD88 to TLR4 followed by the production of pro-inflammatory cytokines [24]. Cytokines-induced activation of nuclear factor-κB (NF-κB) has been introduced in the “Pathway of IκB phosphorylation by complex of receptors MyD88” [23,25]. Not only innate immune but also adaptive immune activation is induced by LPS [26]. A part of the innate immune response are natural killer cells (NK cells) [27,28] that produce IFNγ, just like Th1 cells. Moreover, Th1 cells secrete tumor necrosis factor (TNF), whereas Th2 cells produce IL-4 [29].

Module (c) p50/p65 translocation to the nucleus in M1 macrophages, SMC and EC:

M1 macrophages are answerable to p50/p65 heterodimer. Once p50/p65 NF-κB is translocated into the nucleus, it activates M1 polarization and allows the expression of pro-inflammatory mediators [30].

Module (e) Activation of NF-κB—the canonical pathway via IκBα phosphorylation by IKK (MyD88-dependent signaling pathway):

NF-κB represent a family of transcription factors, present in all eukaryotic cells, that regulate inducible expression of genes involved in immune responses and cell-cycle regulation [31,32]. NF-κB is activated by both the MyD88-dependent and MyD88-independent (TRIF) path upon stimulation of TLR4 by LPS. It leads to phosphorylation and degradation of NF-κB inhibitor by IKK kinases, which allows for translocation of NF-κB to the nucleus.

Module (f) Activation of caspase 1 (by TNF and also by binding with SBE sequences of interferon regulatory factor I (IRF1)):

Caspase 1 plays an important role in the innate immunity and inflammatory diseases as it is essential for the cleavage of pro-IL-1β and pro-interleukin 18 (pro-IL-18) into their mature, biologically active, forms. Activation of caspase 1 is strictly controlled by multi-protein complexes—inflammasomes [33]. The genes encoding caspase 1 are the interferon regulatory factor 1 (IRF1) target genes. IRF1 is activated by many cytokines including IFNs and TNF as well as bacterial components. Initial signaling is mediated through JAK/STAT1 pathway, leading to the activation of the IRF1 promoter by the STAT and NF-κB transcription factors. When a signal is transduced through the IFN receptor, phosphorylated STAT1 translocates into the nucleus where it induces the transcription of primary IFNγ response genes [34].

Module (g) IL-18 synthesis (caspase 1-dependent pathway):

Caspase 1, triggered by stimulation with tumor necrosis factor alpha (TNFα) [35], promotes the conversion of pro-IL-18 into the mature, biologically active, form [36,37].

Module (h) IL-18-IL-18R complex formation:

IL-18 function is mediated through the binding with IL-18 receptor (IL-18R) which contains two subunits: IL-18Rα (ligand binding chain) and IL-18Rβ (signal-transducing chain) [38]. The expression of IL-18R is increased by LPS [39].

Module (i) Negative regulation of IL-18 (by inhibition of caspase 1) caused by nitric oxide (NO):

NO induces S-nitrosylation of NLRP3 and caspase 1 and prevents assembly of the inflammasome [40].

Module (j) NO synthesis:

NO regulates the degree of contraction of vascular smooth muscle cells. In the blood vessel wall, it is mainly produced from L-arginine by endothelial nitric oxide synthase (eNOS), but it can also be released non-enzymatically from S-nitrosothiols or from nitrate/nitrite [41]. Under physiological conditions, the expression of inducible NOS (iNOS) is minimal, but the infection, chronic inflammation and cancer was found to induce it. Its activation requires IRF1 and NF-κB [42]. iNOS continuously produces NO once it is expressed and its expression in the endothelium may contribute to vascular dysfunction by limiting the eNOS action [43].

Module (k) Cardiovascular disease influenced by NO-dependent pathway:

iNOS may have important chronic deleterious effects on the myocardium either caused by increased NO production or, more likely, mediated via peroxynitrite [44].

Module (l) TNF receptor-associated factor 2 (TRAF2) and receptor-interacting serine/threonine-protein kinase 1 (RIP1) ubiquitination:

RIP1 is a dual-function molecule that can be either pro-survival or pro-death depending on its ubiquitination state. In the absence of ubiquitination, RIP1 serves as a pro-apoptotic signaling molecule by engaging caspase 8. It serves as an NF-κB-independent cell death switch early in TNF signaling pathway. TRAF2 is the E3 ligase for RIP1 [45].

Module (m) Macrophages polarization:

The M1/M2 macrophages polarity is triggered by signals present in the surrounding environment. At the most basic level, M1/M2 polarity results from the metabolism of arginine through two pathways: iNOS and arginase. These factors determine which path is dominant, but the surrounding environment determines the final state of macrophage polarization activation. However, after exposure of M2 to M1 signals or vice versa, re-polarization may occur [46,47]. Macrophages play important roles in the organism. In the model, two types of macrophages are distinguished: classical macrophages (M1) and alternatively activated macrophages (M2), which are associated with the Th1 and Th2 polarization [25].

- M1 type (stimulated by IFNγ and TNFα): the main features are the production of pro- inflammatory cytokines: IL-1, 6, 12 and 23. Activation of M1 type is associated with activation of caspase 1 and conversion of pro-IL-18 to active IL-18.
- M2 type: the main feature is the production of an anti-inflammatory cytokine: IL-10. IL-10 enhances the phenotype of M2 macrophages induced by IL-4. IL-4 acts via IL-4 receptor complex which activates STAT6, whereas IL-10 promotes M2 phenotype via activating STAT3 through IL-10 receptor [48].

Module (n) Lipid peroxidation (oxLDL):

NO is engaged in the reduction of superoxide anion radical to peroxynitrite. This mechanism is closely associated with LDL oxidation and production of oxidized low-density lipoprotein (oxLDL) form [49].

Module (o) IL-18 synthesis (caspase 1-independent pathway) and neighboring endothelial cells stimulation:

Synthesis of IL-18 can be mediated by caspase 8, which has been discovered via the more detailed analysis of t-invariants.

Module (p) Regulation of TNFR1 signaling:

TNFR1 has pleiotropic activities such as induction of apoptosis and activation of the transcription factor NF-κB. TNF stimulation induces binding of a TRADD–RIP1–TRAF2–TRAF5 complex to TNFR1. The TRAF proteins then catalyse the polyubiquitination of the kinase RIP1, inducing RIP1 association with the IKK complex (IKK1/IKK2/NEMO) [50]. TRADD-RIP1-TRAF2 complex (complex I) dissociates from TNFR1 and recruits FADD (FAS-associated death domain protein), another TNF effector protein, to form an apoptotic complex (complex II) which activates caspase 8 [51].

Module (r) The role of IL-1, IL-23, IL-6, high level of IL-10 and IL-12 (released by classically activated macrophages M1):

These cytokines are produced by several innate and myeloid immune cells including macrophages and dendritic cells [52]. IL-10 and IL-12 play very important immunoregulatory roles in host defense and immune homeostasis in human organism [53]. IL-23 acts pro-inflammatory and is involved in the differentiation and stabilization of Th17 cells by acting on memory T cells [52,54]. During prolonged and dysregulated exposure to IL-1β and IL-23, Th17 cells recruit inflammatory myeloid cells to cause local tissue inflammation [55]. IL-17 promotes essential hypertension, probably angiotensin II-induced hypertension [56,57].

Module (s) Atherosclerosis progression:

Monocytes attracted by various pro-inflammatory stimuli attach to the inflamed vascular endothelium and penetrate to the arterial intima where they differentiate to macrophages. Intimal macrophages phagocytize oxLDL. Several scavenger receptors mediate oxLDL uptake [58]. Modified LDL are engulfed by the macrophages to form the foam cells. As a result of the accumulation of cholesterol in the foam cells, they break down and form extracellular deposits of cholesterol. The ongoing accumulation of lipoproteins in atherosclerotic lesions leads to their progression and the formation of so-called complex atherosclerotic plaques. They develop in those areas where the severity of fatty streak is greatest. Complex atherosclerotic plaques are characterized by significant fibrosis and coexistence of extracellular cholesterol deposits, which form the so-called plaque lipid core that is filled with cholesterol and necrotic tissue residues. The clinical manifestations of this process depend on the site of the plaque and the occurrence of the thromboembolic phenomena [59,60].

Module (t) Attracting of monocytes (classically activated macrophages M1):

Monocytes, playing a pivotal role in tissue homeostasis, protective immunity, and both promotion and resolution of inflammation are known to originate in the bone marrow from myeloid progenitor cells and then released into the peripheral blood. After 10 to 20 h, they enter into the tissues so as to be activated and differentiated into macrophages [61,62]. Macrophages can acquire distinct phenotypes and biological functions depending on the microenvironment and the metabolic state. Pro-inflammatory stimuli-induced macrophages M1 possess pro-inflammatory features. M1-polarized macrophages mediate resistance to intracellular pathogens and tumors in the context of Th1-driven responses [63].

Module (u) Formation of apoptosome and activation of effector caspases 3, 6, 7:

The mitochondrial “intrinsic” pathway and the death receptor “extrinsic” pathway are the two principal routes leading to apoptosis (the regulated destruction of a cell). Both of these pathways converge on caspase activation. Damage to mitochondria and subsequent apoptosome-mediated caspase 9 activation is accepted as the initiating event in the intrinsic pathway of apoptosis [64]. Caspase 9 can directly activate the effector caspase, i.e., caspase 3 [65].

Module (w) High level of active caspase 8:

The extrinsic pathway of apoptosis is induced by ligand-mediated activation of the TNF family of cell surface receptors [45]. The main death receptors: DR4 and DR5 (TNF-related apoptosis inducing ligand (TRAIL) receptors) and Fas induce cell death following ligation with TRAIL or Fas ligand (FasL), respectively, followed by recruitment of pro-caspase 8 [66]. A large protein complex—receptor/adaptor/caspase-8 death-inducing signaling complex (DISC) forms at the cell membrane and recruits and then activates caspase 8 (initiator caspase). Active caspase 8 directly processes and activates effector caspases. TNF receptor 1 (TNFR1) can trigger opposing responses within the same cell: one leads to a pro-survival response (by activation of the NF-κB signaling pathway), whereas the other leads to cell death (activation of caspase 8 and 3) [67].

Module (x) Inhibition of caspase 8 and inhibition of apoptosis by a high level of cellular FADD-like IL-1β-converting enzyme (FLICE) inhibitory proteins (cFLIPs):

cFLIPs inhibit death receptor-mediated apoptosis by preventing caspase 8 activation [68].

Module (y) STAT6 upregulation promotes M2 macrophage polarization to suppress atherosclerosis:

STAT3 and STAT6 have been found to be potentially involved in regulating the M1 to M2 phenotypic switch [69]. The anti-inflammatory M2 properties, induced by IL-4 or IL-13 are associated with tissue repair and endocytic clearance by secreting anti-inflammatory factors [70]. IL-10 enhances the M2 phenotype induced by IL-4 [2].

The names of places and transitions of the model presented in Figure 5 are included in appendices in Table A1 and Table A2. Here, all of the used shortcuts have been explained. Table A1 includes names of the biological or chemical components corresponding to each place while Table A2 contains biological meanings of the processes corresponding to each transition. Additionally, Table A2 includes “auxiliary transition”. More precisely, in the proposed model, there are two types of auxiliary transitions. The first type is the input transition, which corresponds to the reactions involved in the production of biological or chemical components. The second type of auxiliary transition is the output transition corresponding to the mechanisms engaged in different processes, which are not included in the model because they are not important for the modeled phenomenon.

Classical Petri nets have some limitation, e.g., analysis of Petri net-based model which includes inhibition reactions. In order to model the inhibition reactions, the Snoopy tool allows the use of inhibitor arcs, but they are an extension of classical Petri nets and they are not included in an incidence matrix, which is a base for determination of t-invariants necessary for the analysis of the model. For this reason, a new approach for modeling inhibition reaction using classical Petri nets has been used (this method has been described in greater detail in our paper [71]). This approach is used in our model because these kind of reactions are essential for the modeled biological system since they primarily pertain to the regulation of IL-18 (positive regulation of IL-18 and inhibition of its activity).

## 4. Methods

A Petri net is a mathematical object whose structure is a weighted directed bipartite graph. Such a net consists of two disjoint subsets of vertices, called places and transitions, which can be connected by arcs in such a way that no two places nor two transitions are joined by an arc. When a Petri net is a model of a biological system, places correspond to its passive components, as chemical compounds, while transitions are counterparts of active components, as chemical reactions. Arcs describe causal relations between passive and active components. Places, transitions and arcs constitute the bipartite graph being a structure of a Petri net, but there is another important kind of Petri net components, i.e., tokens. They bring dynamics to the nets, being their crucial property. A distribution of tokens over a set of places called a marking of the net corresponds to a state of the modeled system [10,11,12].

Formally, a Petri net can be defined as 5-tuple $N=(P,T,F,W,M_{0})$, where: $P=\{p_{1},p_{2},\ldots ,p_{n}\}$ is a finite set of places, $T=\{t_{1},t_{2},\ldots ,t_{m}\}$ is a finite set of transitions, F⊆(P×T)∪(T×P) is a set of arcs, $W:F\to Z^{+}$ is a weight function, $M_{0}:P\to N$ is an initial marking, P∩T=∅ and P∪T≠∅ [10].

Tokens flow from one place to another through transitions. The flow of them corresponds to a flow of substances, information, etc. in the modeled system. It is governed by a simple transition firing rule. According to its transition, $t_{j}$ is enabled if in every place $p_{k}$ directly preceding it (called a pre-place of this transition), the number of tokens residing there is equal to at least $w(p_{k},t_{j})$, i.e., the weight of the arc connecting $p_{k}$ with $t_{j}$.

Enabled transition $t_{j}$ can be fired, which means that tokens flow from its pre-places to its post-places, i.e., those ones which directly succeed it, and the number of flowing tokens is equal to the weight of a given arc. There are two exceptions to this rule, i.e., a transition without pre-places, called an input transition, is continuously enabled, and a transition without post-places, called an output transition, when fired, does not produce any tokens [13,72].

Petri nets have an intuitive graphical representation, which is very helpful in understanding its structure and also supports a simulation of a net. However, it is not very well suited for a formal analysis of Petri nets properties. For this purpose, another representation, i.e., an incidence matrix is used. In such matrix, $A=[a_{ij}]$ rows correspond to places while columns correspond to transitions. Entry $a_{ij}$ has a value of $w\left(t_{j},p_{i}\right)-w\left(p_{i},t_{j}\right)$, i.e., it is equal to the difference between the numbers of tokens in place $p_{i}$ after and before firing transition $t_{j}$ (if arc (x,y) does not exist in the net, then w(x,y)=0).

When a Petri net is a model of some biological system, an especially important method of analysis of such a model is based on transition invariants (t-invariants). Such an invariant is vector $x\in N^{m}$, being a solution to the equation A·x=0. For t-invariant *x*, there is a corresponding set of transitions $s\left(x\right)=\left\{t_{j}:x_{j}>0,j=1,2,\ldots ,m\right\}$, called its support. If every transition $t_{j}\in s\left(x\right)$ is fired $x_{j}$ times, then the marking of the net does not change. Usually, the net should be covered by t-invariants, which means that every transition belongs to a support of at least one t-invariant [13,72,73].

While transitions correspond to elementary processes of the modeled system, t-invariants (or more precisely, their supports) are counterparts of subprocesses of a higher level. Since firing every transition from a support of a given t-invariant a proper number of times does not change a marking of the net, t-invariants correspond to subprocesses which do not change a state of the modeled system. Obviously, each of these subprocesses is composed of some elementary processes corresponding to transitions. If two or more subprocesses are composed of some common elementary processes, they can interact with each other via them, which can be a source of some properties of the modeled system. Thus, looking for subsets of elementary processes which are common for some higher level subprocesses can lead to discoveries of some properties of the system. It can be done by looking for similarities between t-invariants (which is equivalent to a similarity between their supports if a proper similarity measure is used) [17,74].

When the number of t-invariants is large, in the process of searching for similarities among them, clustering algorithms are usually used. As a result, a collection of sets, called t-clusters, containing t-invariants similar to each other according to some similarity measure is obtained and looking for meaningful similarities is done within these clusters. In general, clustering of t-invariants is not a trivial task because there is a lot of clustering algorithms and similarity measures and the most suitable ones should be used. However, there are no general rules for selecting the proper algorithm and the similarity measure. Moreover, an appropriate number of clusters should also be determined.

One of the methods for solving this problem is to generate various clusterings (i.e., the sets of clusters) and then evaluate their quality. It can be done using some indices, as Mean Split Silhouette (MSS) and Calinski–Harabasz (C–H) [15,17,18,75].

Moreover, transitions can also be grouped into sets called Maximal Common Transition sets (MCT sets). A set of this type contains transitions which are elements of supports of exactly the same t-invariants. These sets partition the set of all transitions into disjoint subsets and each of them corresponds to some functional block of the modeled biological system. An analysis of MCT sets usually can provide additional information about properties of the studied biological phenomenon [18,76].

**Figure 6:**
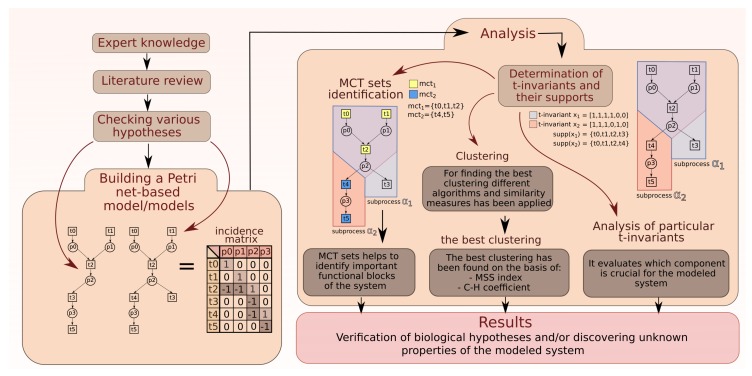
The scheme of work: creating models, methods of analysis, and obtaining results.

The general scheme of work during the process of creating and analysis a Petri net-based model is shown in Figure 6 (through expert knowledge, checking various hypotheses to creating model and analysis based on t-invariants).

## 5. Conclusions

**Figure 7:**
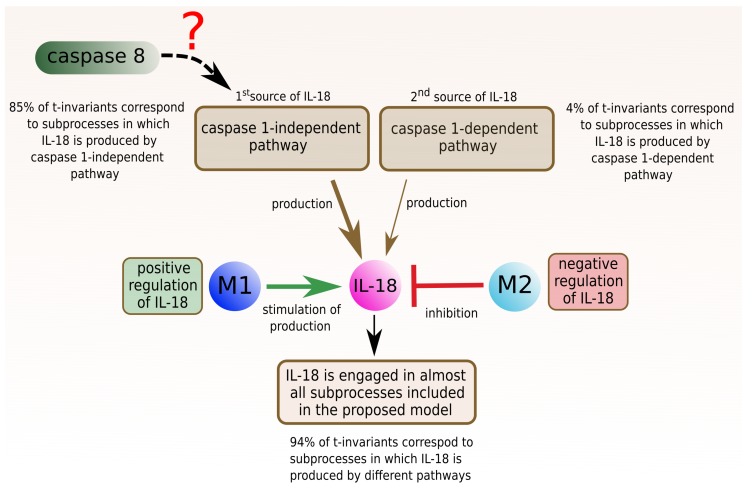
The influence of different types of macrophages on IL-18. The results suggest that caspase 1-independent pathway may be associated with caspase 8 action.

Two types of macrophages occur together in the majority of biological subprocesses in the modeled system. However, in this study, subprocesses in which M1 and M2 macrophages are involved separately have been distinguished. It is important to note that the number of t-invariants corresponding to subprocesses including M1 macrophages (21%) was significantly higher than the number of t-invariants associated with M2 macrophages (6%). In the case of IL-18 the number of t-invariants (94%) indisputably confirmed wide influence of IL-18 on almost all studied subprocesses. It should be underlined that, in our study, interesting properties of dependencies between IL-18 and different types of macrophages have been discovered. We have revealed that M1 macrophages are involved in positive regulation of IL-18, whereas M2 macrophages are involved in negative regulation of this cytokine. However, in the majority of the studied subprocesses, macrophages M1 and M2 occured together. Moreover, the analysis focused on IL-18 allowed for discovering that IL-18 produced in the caspase 1-independent pathway is involved in significantly more subprocesses than IL-18, which is produced in the caspase 1-dependent pathway. In addition, we found that caspase 1-independent pathway may be associated with caspase 8 action. More than half of t-invariants (62.82%) correspond to subprocesses in which caspase 8 occur along with IL-18, which was produced by caspase-1 independent pathway. Conclusions mentioned above have been presented in Figure 7.

## Figures and Tables

**Table 1:** List of non-trivial Maximal Common Transition (MCT) sets. The column “MCT set” contains the names of MCT sets. The column “Contained transitions” includes names of transitions contained in a given MCT set. The column “Biological interpretation” includes biological description (functions and mechanisms) of MCT sets.

MCT Set	Subprocesses from	Contained Transitions	Biological Interpretation
$m_{1}$	d, e, h	$t_{1}$, $t_{2}$, $t_{16}$, $t_{17}$, $t_{18}$, $t_{19}$, $t_{20}$, $t_{21}$, $t_{22}$, $t_{29}$, $t_{41}$	IL-18R-mediated Myd88 signaling pathway.
$m_{2}$	u	$t_{44}$, $t_{46}$, $t_{47}$, $t_{48}$, $t_{52}$, $t_{53}$, $t_{54}$	Intrinsic pathway of apoptosis. This pathway arises from signals that originate within the cell and is associated with BID cleavage via radiation, toxins or hypoxia. Truncated BID translocates to mitochondria and is involved in the release of active APAF1 and cytochrome c from mitochondria to the cytosol. Active APAF1 and cytochrome c create a complex, which leads to triggering the formation of apoptosome and activation of caspase 9. Active caspase (textcolorredinitiator caspase) initiates apoptosis by cleaving and thereby activating executioner caspases.
$m_{3}$	b, e	$t_{12}$, $t_{13}$, $t_{15}$, $t_{71}$, $t_{72}$, $t_{73}$	Recognition of lipopolysaccharide pattern by TLR4 complexes. Activated TL4 is engaged on TLR and MyD88 connection via TIRAP.
$m_{4}$	c, e	$t_{14}$, $t_{23}$, $t_{45}$, $t_{89}$, $t_{90}$	Activation of NF-κB - the canonical pathway via IκBα phosphorylation by IKK (MyD88-dependent signaling pathway). Translocation of p50-p65 to the nucleus in M1 macrophages, SMC and EC.
$m_{5}$	y, r	$t_{59}$, $t_{60}$, $t_{61}$, $t_{62}$, $t_{76}$	IL-4–induced M2 polarization. Firstly, STAT3 activated by IL-6-IL6-R-gp130complex, influenced by high level of IL-10, induces binding IL-4 with type I IL-4R, which results in STAT6 pathway activation.
$m_{6}$	k, n	$t_{39}$, $t_{40}$, $t_{42}$, $t_{43}$	High shear stress (mediated by ROS generation) and cardiac contractile dysfunction (affected by nitric oxide) lead to CVD.
$m_{7}$	r	$t_{78}$, $t_{79}$, $t_{80}$, $t_{81}$	A lot of classically activated macrophages M1 release IL-1 and IL-23, which influence Th17 formation. Th17 cells are engaged in essential hypertension.
$m_{8}$	g	$t_{0}$, $t_{30}$, $t_{95}$	Classically activated macrophages M1, under influence of an inflammatory microenvironment, induce IL-18 synthesis via cleavage of pro-IL-18 by caspase-1.
$m_{9}$	p	$t_{5}$, $t_{7}$, $t_{58}$	SODD binds to TNFR1 and prevents self-aggregation and spontaneous downstream signaling at ligand absent (TNFR1 stabilization). SODD dissociates from TNFR1 upon receptor ligation. Binding of trimeric TNFα to TNFR1 induces receptor trimerization and recruitment of TNFR1-associated death domain protein (TRADD), which serves as a platform to recruit next additional mediators. Active TNFR1 signaling complex is engaged on the formation of TRAF2-TRADD-RIP1-TRAF5-TNFR1 complex.
$m_{10}$	p	$t_{25}$, $t_{36}$, $t_{88}$	Internalization of TNFR1 triggers pro-apoptotic signals (via the FAS-associated death domain adapter protein (FADD)).
$m_{11}$	l	$t_{26}$, $t_{27}$, $t_{35}$	TRAF2-TRADD-RIP1-TRAF5-TNFR1 complex and cFLIP lead to TRAF2 ubiquitination and next to RIP1 ubiquitination, which recruits TAK1 (via TAB2).
$m_{12}$	a, f	$t_{37}$, $t_{38}$, $t_{87}$	JAK-STAT pathway stimulated by IFNγ. Activation pathway of JAK1, JAK2 and STAT1, leads to binding with SBE sequences of IRF1. IRF-1 binds to the IFN regulatory factor element (IRF-E) present in the inducible NO synthase (iNOS) promoter, and together with NF-κB and STAT1, activate its transcription. This process leads to NO synthesis.
$m_{13}$	i	$t_{55}$, $t_{69}$, $t_{70}$	Nitric oxide leads to caspase 1 inhibition by S-nitrosylation of cysteine residues at the active sites. Negative regulation of IL-18 synthesis.
$m_{14}$	y	$t_{64}$, $t_{65}$, $t_{77}$	Processes leading to atherosclerosis plaque regression have an influence on increasing anti-inflammatory pathways, which results in inhibition of M1 macrophage specific gene expression and alternatively in promotion of M2 macrophage specific gene expression.
$m_{15}$	y	$t_{67}$, $t_{82}$, $t_{83}$	TWEAK–CD163-expressing M2 macrophages interaction.
$m_{16}$	w	$t_{8}$, $t_{28}$	TRAIL or FasL bind their cognate receptors which induces receptor trimerization and formation of the death inducing signaling complex (DISC), type I cells comprising FADD and caspase-8.
$m_{17}$	n	$t_{9}$, $t_{86}$	Peroxynitrite formation catalyzed by NADPH oxidase.
$m_{18}$	t	$t_{33}$, $t_{96}$	Attracting of monocytes (influenced by TRAIL).
$m_{19}$	x	$t_{49}$, $t_{51}$	Increased quantity of cFLIP can inhibit DISC formation by competing with caspase-8/10 for binding to FADD.
$m_{20}$	t	$t_{56}$, $t_{57}$	TRAIL and its cognate receptors: death receptors (DR5 and DR4) trimerization.
$m_{21}$	m	$t_{74}$, $t_{75}$	Transformation of tissue macrophages to M1 (a lot of classically activated macrophages M1).
$m_{22}$	c	$t_{93}$, $t_{94}$	The heterodimer p50-p60 translocation to the nucleus in M1 macrophages, SMC and EC where it binds to specific κB sites and activates a variety of NF-κB target genes, including VCAM1 and ICAM1.

**Table 2:** List of t-clusters. The column “t-cluster” contains the names of t-clusters. The column “Biological meaning” includes biological interpretations of t-clusters.

t-Cluster	Biological Meaning
$c_{1}$	TRAIL-induced apoptosis signaling pathways. Increased quantity of cFLIP leads to inhibition of caspase 8.
$c_{2}$	The mitochondria-involved intrinsic apoptotic pathway. The intrinsic pathway arises from signals that originate within the cell, as a consequence of cellular stress or DNA damage. High level of IL-10 has an influence on development of anti-inflammatory processes. Damage to mitochondria and subsequent apoptosome-mediated caspase 9 activation, which directly activate the effector caspase, caspase 3.
$c_{3}$	The mitochondria-involved intrinsic apoptotic pathway, similar to $c_{3}$, accompanied by cleavage and activation of caspases 3, 6 and 7.
$c_{4}$	TWEAK leads to the reaction catalyzed by NADPH oxidase, which results in peroxynitrite production. Macrophages M2 cause TWEAK sequestration in case of high level of IL-10. Peroxynitrite is engaged in lipids peroxidation and results in the production of modified oxidized LDL. This modified oxidized LDL together with IL-18 lead to neighboring endothelial cell stimulation and secretion of MCP1 (IL-18 is produced in caspase 1-independent pathway).
$c_{5}$	This cluster contains almost all processes included in the model, however it is missing: Caspase 1 inhibition caused by NO. TNFR1 endocytosis from TRAF2-TRADD-RIP1-TRAF5 complex, which leads to omission of FADD. Damage to mitochondria and subsequent apoptosome-mediated caspase 9 activation.
$c_{6}$	Attracting of monocytes caused by MCP1, VCAM1, ICAM1 (secreted via p50/p65 translocation to the nucleus in macrophages M1 and SMC and EC) and TRAIL (in oncogenesis) lead to a lot of classically activated macrophages M1. In oncogenesis TRAIL and death receptors trimerization leads to pro-caspase 8 recruitment by FADD. Connection through DDs within FADD and TNFR result in active DISC type I cells and increased quantity of cFLIP. cFLIP together with TRAF2-TRADD-RIP1-TRAF5-TNFR1 complex lead to TRAF2 ubiquitination and in consequence to RIP1 ubiquitination. RIP1 recruits TAK1 via TAB2, which is part of IKK complex engaged in phosphorylation of IκB (which stimulate p50/p65 translocation to the nucleus in macrophages M1, SMC and EC). p50/p65 translocation to the nucleus in macrophages M1 and SMC and EC leads to secretion of iNOS, which is associated with NO synthesis. NO synthesis leads also to peroxynitrite production, which together with high level of caspase 8 result in activation of caspases 3, 6 and 7. The high level of caspase 8 is caused by TRAIL and TRAIL death receptors trimerization (in oncogenesis). Active caspases 3, 6, 7 and also TWEAK lead to apoptosis enhancement anti-inflammation. TWEAK sequestration is caused by macrophages M2 in case of high level of IL-10. Moreover, NO is engaged in cardiac contractile dysfunction, which leads to CVD symptoms and cardiovascular events. These symptoms are additionally stimulated by ROS generation and foamy cells, which are associated with NO synthesis.
$c_{7}$	This cluster contains almost all processes included in the model; however, it is missing: Caspase 1 inhibition caused by NO.
$c_{8}$	This cluster contains almost all processes included in the model, however it is missing: IL-18 synthesis caused by caspase 1-dependent pathway. However, in this cluster IL-18 is produced in caspase 1-independent pathway. IL-12, IL-1, IL-23 release by classically activated macrophages M1. IFN gamma synthesis, what results in omission of JAK1, JAK2 and STAT1, STAT2 activation and also STAT1 protein via IFN gamma and IFNR. Omission of JAK- STAT1 pathway activation results in a lack of caspase 1 (a lack of modulation by TNF is also present). TNFR1 trimerization, which can create complex with TRAF2-TRADD-RIP1-TRAF5, which leads to the omission of FAD recruitment and TRAF2 ubiquitination.
$c_{9}$	This cluster contains almost all processes included in the model, however it is missing: IL-18 synthesis caused by caspase 1-dependent pathway. However, in this cluster IL-18 is produced in caspase 1-independent pathway. IL-12, IL-1, IL-23, IL-6 release by classically activated macrophages M1. High level of IL-10 release by alternatively activated macrophages M2. IFN gamma synthesis, which results in omission of JAK1, JAK2 and STAT1, STAT2 activation and also STAT1 protein via IFN gamma and IFNR. Omission of JAK- STAT1 pathway activation results in a lack of caspase 1 (a lack of modulation by TNF is also present). Caspase 8 inhibition.
$c_{10}$	Attracting of monocytes stimulated by TRAIL, MCP1 (by neighboring endothelial cells stimulation) and MCP1, VCAM1, ICAM1 (by p50/p65 translocation to the nucleus in macrophages M1 and SMC and EC) leads to high level of classically activated macrophages M1, which release IL-6, IL-12, IL-1, IL-23. Macrophages M1 are also engaged in the transformation of oxLDL into foamy cells via NO synthesis and lipid peroxidation, which results in progression of atherosclerotic plaque. IL-18 is produced in caspase 1-independent pathway and it can create complex with IL-18R (IL-18R is stimulated by bacterial infections). Active IL-18-IL-18Rα-IL18Rβ complex recruits MyD88 which is engaged in a pathway of IκB phosphorylation. IκB phosphorylation stimulates attract of monocytes via p50/p65 translocation to the nucleus in macrophages M1 and SMC and EC, which stimulate MCP1, VCAM1, ICAM1, TNF alpha, iNOS. IL-1 and IL-23 induce Th17 differentiation. Th17 cells are engaged in essential hypertension. IL-6 and high level of IL-10 lead to activation of STAT3 protein, which is engaged in an induction of IL-4 receptor alpha and binding with IL-4. IL-4 and IL-4R complex stimulates anti-inflammatory pathway, which in consequence leads to high level of IL-10. This cluster includes also negative regulation of IL-18. To be precise, IFN gamma leads to interaction with IFNRs, which results in JAK1, 2 and STAT1, STAT2 activation. This activation leads to binding with SBE sequences of IRF1, which result in NO synthesis induced by iNOS. It is important that NO is engaged in caspase 1 inhibition and in consequence leads to negative regulation of IL-18.
$c_{11}$	Attracting of monocytes stimulated by TRAIL, MCP1, VCAM1, ICAM1 (by p50/p65 translocation to the nucleus in macrophages M1 and SMC and EC) lead to high level of classically activated macrophages M1, which release IL-6, IL-12, IL-1, IL-23. IL18 is produced in caspase 1-independent pathway and it can create complex with IL-18R (IL-18R is stimulated by bacterial infections). Active IL-18-IL-18Rα-IL-18Rβ complex recruits MyD88 which is engaged in a pathway of IκB phosphorylation. Moreover, receptor MyD88 complex can be additionally stimulated by LPS-LBP complex (TLR4 and MyD88 connection via TIRAP). IκB phosphorylation stimulates attracting of monocytes via p50/p65 translocation to the nucleus in macrophages M1 and SMC and EC, which stimulate MCP1, VCAM1, ICAM1, TNF alpha, iNOS. IL-1 and IL-23 lead to the formation of Th17. Th17 cells are engaged in essential hypertension. IL-6 and high level of IL-10 lead to activation of STAT3 protein, which is engaged in an induction of IL-4 receptor alpha and binding with IL-4. IL-4 and IL-4R complex stimulates anti-inflammatory pathway, which in consequence leads to high level of IL-10. This cluster includes also negative regulation of IL-18. To be precise, IFN gamma leads to interaction with IFNRs, which results in JAK1, 2 and STAT1, STAT2 activation. This activation leads to binding with SBE sequences of IRF1, which result in NO synthesis induced by iNOS. Important is fact, that NO is engaged in caspase 1 inhibition and in consequence leads to negative regulation of IL-18. Binding of TRAIL to death receptors (DR5 and DR4) leads to the recruitment of an adaptor protein FADD, which leads to pro caspase 8 recruitment. The connection through DDs within FADD and TNFR leads to an activation of DISC type I cells. High level of caspase 8 together with peroxynitrite production result in activation of caspases 3, 6 and 7. Active caspases 3, 6, 7 and also TWEAK lead to apoptosis enhancement anti-inflammation.
$c_{12}$	Attract of monocytes stimulated by TRAIL, MCP1 (by neighboring endothelial cells stimulation) and MCP1, VCAM1, ICAM1 (by p50/p65 translocation to the nucleus in macrophages M1 and SMC and EC) leads to high level of classically activated macrophages M1, which release IL-6, IL-12, IL-1, IL-23. Macrophages M1 are also engaged in the transformation of oxLDL into foamy cells via NO synthesis and lipid peroxidation, which results in progression of atherosclerotic plaque. IL-18 is produced in caspase 1-independent pathway and it can create complex with IL-18R (IL-18R is stimulated by bacterial infections). Active IL-18-IL-18Rα-IL-18Rβ complex recruits MyD88 which is engaged in a pathway of IκB phosphorylation. IκB phosphorylation stimulates attract of monocytes via p50/p65 translocation to the nucleus in macrophages M1 and SMC and EC, which stimulates MCP1, VCAM1, ICAM1, TNF alpha and iNOS. IL-1 and IL-23 lead to the formation of Th17. Th17 cells are engaged in essential hypertension. IL-6 and high level of IL-10 lead to an activation of STAT3 protein, which is engaged in an induction of IL-4 receptor alpha and binding with IL-4. IL-4 and IL-4R complex stimulates anti-inflammatory pathway, which in consequence, leads to high level of IL-10. Binding of TRAIL to death receptors (DR5 and DR4) leads to the recruitment of an adaptor protein FADD, which leads to pro caspase 8 recruitment. The connection through DDs within FADD and TNFR leads to an activation of DISC type I cells. Increased quantity of cFLIP leads to inhibition of caspase 8.
$c_{13}$	IFN gamma leads to interaction with IFNRs, which results in JAK1, JAK2 and STAT1, STAT2 activation. Activation of pathway of JAK-STAT1 leads to binding with SBE sequences of IRF-1. This process leads to NO synthesis induced by iNOS. NO is engaged in caspase 1 inhibition and in consequence leads to negative regulation of IL-18. NO synthesis leads also to peroxynitrite production, which together with high level of caspase 8 results in an activation of caspases 3, 6 and 7. High level of caspase 8 is caused by TRAIL and TRAIL death receptors trimerization (in oncogenesis). Active caspases 3, 6, 7 and also TWEAK lead to apoptosis enhancement anti-inflammation. TWEAK sequestration is caused by macrophages M2 in case of high level of IL-10.
$c_{14}$	IFN gamma leads to interaction with IFNRs, which results in JAK1, 2 and STAT1, STAT2 activation. Activation of pathway of JAK-STAT1 leads to binding with SBE sequences of IRF1. This process leads to NO synthesis induced by iNOS. NO is engaged in caspase 1 inhibition and in consequence leads to negative regulation of IL-18. NO synthesis leads also to peroxynitrite production, which is engaged in lipid peroxidation and results in production of modified oxLDL. This modified oxLDL together with IL-18 lead to neighboring endothelial cell stimulation and secretion of MCP1 (IL-18 is produced in caspase 1-independent pathway).
$c_{15}$	This cluster contains almost all processes included in the model, however it is missing: IL-18 synthesis caused by caspase 1-dependent pathway. However, in this cluster IL-18 is produced in caspase 1-independent pathway. Increased quantity of cFLIP, which results in omission of caspase 8 inhibition (and apoptosis inhibition) and omission of ubiquitination of TRAF2 and RIR1. Classically activated macrophages M1 via transformation of tissue macrophages. In this case, IL-1, IL-23 and L-12 are not released. IL-1 and IL-23 are engaged in Th17 cell formation, which leads to essential hypertension. IL-12 is engaged in IFN gamma synthesis. In this cluster, IFN gamma synthesis is caused by LPS (bacterial infection).

**Table 3:** Comparison of t-clusters analysis and the more detailed analysis of particular t-invariants in terms of occurring of IL-18 and macrophages in subprocesses.

	Analysis of t-Clusters	The More Detailed Analysis of Particular t-Invariants
	Subprocesses in Which IL-18 Is Engaged	
**Sources of IL-18:**	**Number of t-Clusters**	**Number of t-Invariants**
Caspase 1-independent pathway	10 (67%)	66 (85%)
Caspase 1-dependent pathway	0 (0%)	3 (4%)
Caspase 1-independent pathway and caspase 1-dependent pathway	2 (13%)	4 (5%)
lack of IL-18	3 (20%)	5 (6%)
	**Subprocesses in Which Macrophages Are Engaged**	
**Type of Macrophages:**	**Number of t-Clusters**	**Number of t-Invariants**
M1	1 (7%)	16 (21%)
M2	4 (27%)	5 (6%)
M1 and M2	8 (53%)	54 (69%)
lack of macrophages	2 (13%)	3 (4%)

## Appendix A. List of Places

**Table A1:** List of places. The column “No.” includes the names of places and the column “Biological meaning” includes the names of biological or chemical components corresponding to places.

No.	Biological Meaning	No.	Biological Meaning
$p_{0}$	active apoptotic peptidase activating factor 1 (APAF1) [77]	$p_{42}$	foamy cells [49]
$p_{1}$	apoptotic protease activating factor 1 (APAF1) and cytochrome c complex [77]	$p_{43}$	high level of active caspase 8 active in type I cells [78]
$p_{2}$	FAS-associated protein with death domain (FADD) recruited [78]	$p_{44}$	high shear stress [49]
$p_{3}$	large amounts of cell-surface FAS receptor (FAS) and FAS ligand (FASL) [78]	$p_{45}$	inducible nitric oxide synthase (iNOS) [49]
$p_{4}$	intercellular adhesion molecule 1 (ICAM1)	$p_{46}$	low level of active caspase 8 in type I cells
$p_{5}$	interferon gamma (IFNγ) and interferon receptors (IFNRs) complex [79]	$p_{47}$	modified, oxidized LDL [49]
$p_{6}$	kinase complex (IKK complex made of two kinase IKKα and IKKβ and regulatory subunit NF-κB essential modulator (NEMO) also known as inhibitor of nuclear factor κ-B kinase subunit gamma (IKKγ) [80]	$p_{48}$	nitric oxide (NO) [49]
$p_{7}$	interleukin 18 (IL-18) [25,35,37]	$p_{49}$	p50/p65 proteasome inhibitors induce inhibitory κB (IκB) phosphorylated complex within macrophages [80]
$p_{8}$	interleukin 18 receptor (IL-18R) [38]	$p_{50}$	p50/p65 dimer nuclear factor kappa-light-chain-enhancer of activated B cells (NF-κB) early phase in activated endothelial cell (EC) in atherosclerosis [80]
$p_{9}$	IRAK1P-TRAF6-TAB2-TAB1-TAK1 complex at plasma membrane, where: IRAK1P—interleukin 1 receptor-associated kinase 1 protein, TRAF6—TNF receptor associated factors 6 (TNF—tumor necrosis factor), TAB1 and TAB2—transforming growth factor (TGFβ) activated kinase 1 binding protein 1 and binding protein 2, TAK1—transforming growth factor (TGFβ) activated kinase 1.	$p_{51}$	p50-p65 dimer nuclear factor kappa-light-chain-enhancer of activated B cells (NF-κB) early phase in activated smooth muscle cell (SMC) [81]
$p_{10}$	interleukin 1 receptor-associated kinase 1 (IRAK1) degraded	$p_{52}$	peroxynitrite [49]
$p_{11}$	Janus kinases and signal transducer and activator of transcription 1 (JAK/STAT1) pathway activated	$p_{53}$	pro-interleukin 18 (pro-IL18) [25,37]
$p_{12}$	lipopolysaccharide binding protein (LBP) [23,82]	$p_{54}$	pro-caspase 8 recruited
$p_{13}$	lipopolysaccharide (LPS) [23,25,26,39,82]	$p_{55}$	radiation and/or toxins and/or hypoxia
$p_{14}$	lipopolysaccharide (LPS) and lipopolysaccharide binding protein (LBP) complex [23,82]	$p_{56}$	truncated BH${}_{3}$ interacting-domain death agonist (BID)
$p_{15}$	monocyte chemotactic protein 1 (MCP1)	$p_{57}$	truncated BID in mitochondria
$p_{16}$	myeloid differentiation primary response gene 88 (MyD88) [23,25]	$p_{58}$	signal transducer and activator of transcription 3 (STAT3) activated proteins [25]
$p_{17}$	receptor-interacting protein 1 (RIP1) ubiquitinated	$p_{59}$	p50-p65 proteasome inhibitors induce inhibitory κB (IκB) phosphorylated complex in activated smooth muscle cell (SMC)[80]
$p_{18}$	receptors myeloid differentiation primary response gene 88 (MyD88) complex [23,25]	$p_{60}$	interleukin 4 (IL-4) [25]
$p_{19}$	TGF-beta activated kinase 1 (TAK1) activated	$p_{61}$	classically activated macrophages M1 [25]
$p_{20}$	toll-like receptor 4 (TLR4) activated [23,82]	$p_{62}$	a lot of classically activated macrophages M1 [25]
$p_{21}$	TLR-MyD88-IRAK4-IRAK1P-TRAF6 complex, where: TLR—toll-like receptor, MyD88—myeloid differentiation primary response gene 88, IRAK4—interleukin-1 receptor-associated kinase 4, IRAK1P—interleukin 1 receptor-associated kinase 1 protein, TRAF6—TNF receptor associated factors 6 (TNF—tumor necrosis factor).	$p_{63}$	high level of interleukin 12 (IL-12) [25]
$p_{22}$	TLR-MyD88-IRAK4-IRAK1P complex	$p_{64}$	no inflammation
$p_{23}$	TLR-Myd88-IRAK4 complex	$p_{65}$	active caspases 3, 6 and 7 [77]
$p_{24}$	tumor necrosis factor receptor 1 (TNFR1) endocytosed	$p_{66}$	TNF-related weak inducer of apoptosis (TWEAK) changed concentration [83]
$p_{25}$	tumor necrosis factor receptor 1 (TNFR1) stable with silencer of death domains (SODD) [81]	$p_{67}$	high level of interleukin 10 (IL-10) [25]
$p_{26}$	tumor necrosis factor alpha (TNFα) [35]	$p_{68}$	subpopulation of activated T cells, T helper 2 (Th2) cells and cluster of differentiation (CD4) [29]
$p_{27}$	TRAF2-TRADD-RIP1-TRAF5-TNFR1 complex, where: TRAF2 and TRAF5-TNF receptor associated factors 2 and 5, TNFR1—tumor necrosis factor receptor 1, TRADD—TNFR1-associated death domain protein, RIP1—receptor-interacting protein 1.	$p_{69}$	inactive caspase 1 [84]
$p_{28}$	TNF receptor associated factors 2 (TRAF2) autoubiquitinated	$p_{70}$	lack of interleukin 18 (IL-18) [84]
$p_{29}$	TNF-related apoptosis-inducing ligand (TRAIL)	$p_{71}$	cluster of differentiation (CD) 4${}^{+}$ T helper cells (Th1) and CD8 effector T cells (CD8${}^{+}$ cytotoxic T cells) [29]
$p_{30}$	TNF-related apoptosis-inducing ligand (TRIAL) death receptors DR5 and DR4	$p_{72}$	natural killer (NK) cells [27,28]
$p_{31}$	UBC13-UEV1A-TRAF6-TAK1P-TAB2P-TAB1 complex, where: UBC13 and UEV1A—ubiquitin-conjugating enzyme complex, TRAF6—TNF receptor associated factors 6, TAB2P—TGFβ activated kinase 1 binding protein 2, TAB1—TGFβ activated kinase 1 binding protein 1.	$p_{73}$	tissue macrophages
$p_{32}$	vascular cell adhesion molecule 1 (VCAM1)	$p_{74}$	interleukin 6 (IL-6) [25]
$p_{33}$	active death-inducing signaling complex (DISC) type I cells [78]	$p_{75}$	development of anti-inflammatory processes
$p_{34}$	active IL-18 and IL-18 receptors (IL-18Rα and IL-18Rβ) complex [38,85]	$p_{76}$	interferon gamma (IFNγ)
$p_{35}$	active TNF receptor associated factors 1 (TNFR1) signaling complex	$p_{77}$	interleukin 1 β (IL-1β) [25,86,87]
$p_{36}$	apoptosome [77]	$p_{78}$	interleukin 23 (IL-23) [25,86,87]
$p_{37}$	cellular FLICE (FADD-like IL-1β-converting enzyme)-inhibitory protein (cFLIP) [78]	$p_{79}$	cytokines secreted by Th17 cells (IL-17, IL-21, and IL-22) [87,88]
$p_{38}$	cardiovascular disease [49]	$p_{80}$	human regulatory T cells
$p_{39}$	interleukin 1β converting enzyme (ICE)/caspase 1 protease [37]	$p_{81}$	NADPH oxidase [89]
$p_{40}$	contractility failure [49]	$p_{82}$	interleukin 4 (IL-4) and interleukin 4 receptor (IL-4R) complex [25]
$p_{41}$	cytochrome c [77]	$p_{83}$	alternatively activated macrophages M2 with scavenger receptor A (SRA1) and cluster of differentiation 163 (CD163) [25]

## Appendix B. List of Transitions

**Table A2:** List of transitions. The column “No.” includes the names of transitions and the column “Biological meaning” includes the names of biological functions of transition.

No.	Biological Meaning	No.	Biological Meaning
$t_{0}$	pro-interleukin 18 (pro-IL-18) activation by caspase 1 enzymatic cleavage [36,90]	$t_{49}$	inhibition of active caspase 8 in type I cells
$t_{1}$	IL-18 binding with IL-18R receptor [91]	$t_{50}$	increase quantity of cFLIP [92,93]
$t_{2}$	bacterial infection	$t_{51}$	apoptosis inhibition [91,93]
$t_{3}$	caspase 8 dimerization autocleavage and activation type I cells (cells in which caspase-8 activation is sufficient to induce apoptosis) [67,92]	$t_{52}$	intrinsic pathway of apoptosis [92,94]
$t_{4}$	cleavage and activation of caspases 3, 6 and 7	$t_{53}$	cytochrome c binding to apoptotic protease activating factor 1 (APAF1) [94]
$t_{5}$	tumor necrosis factor receptor 1 (TNFR1) trimerization [81]	$t_{54}$	triggering the formation of apoptosome [94]
$t_{6}$	nitric oxide synthesis	$t_{55}$	caspase 1 inhibition [84]
$t_{7}$	forming TRAF2-TRADD-RIP1-TRAF5-TNFR1 complex [81], where: TRAF2 and TRAF5- tumor necrosis factor (TNF) receptor associated factors 2 and 5, TNFR1 - TNF receptor 1, TRADD - TNFR1-associated death domain protein, RIP1 - receptor-interacting protein 1.	$t_{56}$	trimerization of TNF-related apoptosis-inducing ligand (TRIAL) death receptors [93]
$t_{8}$	pro-caspase 8 recruitment influenced by FADD [93,95]	$t_{57}$	oncogenesis/inflammation [93]
$t_{9}$	NADPH oxidase activity enhancement by TWEAK [83,89]	$t_{58}$	silencer of death domains (SODD) binds to tumor necrosis factor receptor 1 (TNFR1) leading to its stabilization [81]
$t_{10}$	lipids peroxydation	$t_{59}$	activation of signal transducer and activator of transcription 3 (STAT3) proteins phosphorylation by JAK via interleukin 6, interleukin 6 receptor and glycoprotein 130 complex (IL-6-IL-6R-gp130) on target cells (classical signaling pathway) [96]
$t_{11}$	transformation into foamy cells	$t_{60}$	interleukin 4 receptor (type I IL-4R)) activation via binding with interleukin 4 (IL-4)and JAK1/JAK3 phosphorylation [97]
$t_{12}$	binding lipopolysaccharide (LPS) and lipopolysaccharide binding protein (LBP) [82,98]	$t_{61}$	signal transducer and activator of transcription 6 (STAT6) anti-inflammatory pathway [97]
$t_{13}$	binding lipopolysaccharide (LPS) presentation to toll-like receptor 4 (TLR4) and cluster of differentiation 14 (CD14) and myeloid differentiation primary response 2 (Myd2) [82,98]	$t_{62}$	IL-4 synthesis induced by M2 macrophages
$t_{14}$	p50-p65 translocation to the nucleus in macrophages M1, smooth muscle cell (SMC) and endothelial cell (EC) [80]	$t_{63}$	release of interleukin 12 (IL-12) by classically activated macrophages M1
$t_{15}$	toll-like receptor 4 and myeloid differentiation primary response 88 (TLR4-MyD88) connection via toll-interleukin 1 receptor (TIR) domain containing adaptor protein (TIRAP) [98]	$t_{64}$	inhibition of M1 macrophages specific gene expression [47]
$t_{16}$	interleukin-1 receptor-associated kinase 4 (IRAK4) recruitment [91]	$t_{65}$	apoptosis enhancement and anti-inflammatory response [47]
$t_{17}$	connection with interleukin-1 receptor-associated kinase 1 (IRAK1) and its phosphorylation [91]	$t_{66}$	interferon gamma (INFγ) synthesis via IL-12
$t_{18}$	TNF receptor associated factors 6 (TRAF6) recruitment and binding to interleukin 1 receptor- associated kinase 1 protein (IRAK1) [91]	$t_{67}$	TNF-like weak inducer of apoptosis (TWEAK) sequestration
$t_{19}$	dissociation of interleukin 1 receptor-associated kinase 1 protein (IRAK1P) and TNF receptor associated factors 6 (TRAF6) [91]	$t_{68}$	Interferon gamma (INFγ) via IL-12 and IL-12 receptors (IL-12Rs) complexes through signal transducer and activator of transcription 3 and 4 (STAT3 and STAT4)
$t_{20}$	phosphorylation of transforming growth factor (TGFβ) activated kinase 1 and 2 (TAK1 and TAK2) [91]	$t_{69}$	IL-18 negative regulation [84]
$t_{21}$	TRAF6-TAK1P-TAB2P-TAB1 complex translocation to the cytosol [81,91]. Complex, where: TRAF6—tumor necrosis factor (TNF) receptor associated factors 6, TAK1P—transforming growth factor (TGFβ) activated kinase 1, TAB2P—transforming growth factor (TGFβ) activated kinase 1 binding protein 2, TAB1—transforming growth factor (TGFβ) activated kinase 1 binding protein 1.	$t_{70}$	no action of IL-18 [84]
$t_{22}$	TNF receptor-associated factor 6 (TRAF6) ubiquitination [91]	$t_{71}$	adaptive immune response [98]
$t_{23}$	phosphorylation of IKK-β - activation of NF-κB - the canonical pathway [80]	$t_{72}$	innate immune response [98]
$t_{24}$	connection of active IL-18, IL-18R α and β and also myeloid differentiation primary response 2 (IL-18-IL-18Rα-IL-18Rβ-MyD88)	$t_{73}$	interferon gamma (IFNγ) synthesis
$t_{25}$	tumor necrosis factor receptor 1 (TNFR1) clathrin-dependent internalization [99]	$t_{74}$	transformation of tissue macrophages into M1 [25]
$t_{26}$	receptor-interacting protein 1 (RIP1) ubiquitination	$t_{75}$	normal state [25]
$t_{27}$	receptor-interacting protein 1 (RIP1) recruits transforming growth factor β activated kinase 1 (TAK1) via transforming growth factor β activated kinase 1 binding protein 2 (TAB2)	$t_{76}$	release of interleukin 6 (IL-6) by classically activated macrophages M1 [96]
$t_{28}$	connection through death domains (DDs) within FAS-associated protein with death domain (FADD) [93,95]	$t_{77}$	processes leading to atherosclerotic plaque regression [47]
$t_{29}$	recruitment of myeloid differentiation primary response gene 88 (MyD88)	$t_{78}$	T helper 17 (Th17) differentiation [86,88]
$t_{30}$	pro-interleukin 18 (pro-IL-18) synthesis citeDNK+13	$t_{79}$	release of interleukin 1 β (IL-1β) by classically activated macrophages M1 [87,88]
$t_{31}$	atherosclerotic plaque progression	$t_{80}$	release of interleukin 23 (IL-23) by classically activated macrophages M1 [87,88]
$t_{32}$	modulation by TNF	$t_{81}$	autoimmune inflammation, essential hypertension [87,88]
$t_{33}$	attraction of monocytes	$t_{82}$	immune environment changes that stimulate human regulatory T cells (Treg)
$t_{34}$	neighboring endothelial cells stimulation	$t_{83}$	production of IL-10 by Treg
$t_{35}$	TNF receptor-associated factor 2 (TRAF2) ubiquitination	$t_{84}$	IL-10 enhances the M2 phenotype induced by IL-4 [2]
$t_{36}$	recruitment of FAS-associated protein with death domain (FADD) [99]	$t_{85}$	enhancement of M2 effector function
$t_{37}$	interferon gamma (IFNγ) and interferon receptor γ (IFNGR1 and IFNGR2) binding followed by interaction between IFNRs subunits and JAK 1 (IFNGR1) and JAK2 (IFNGR2) what activates latent cytoplasmic STAT1 [100]	$t_{86}$	NADPH oxidase activity source [89]
$t_{38}$	oligomerization of the IFNγ receptor and activation via trans-phosphorylation of the receptor-associated Janus kinases 1 and 2 (JAK1 and JAK2) followed by STAT 1 activation [79,100]	$t_{87}$	binding STAT1 with STAT binding element sequences of interferon regulatory factor 1 (IRF1) in IFNγ pathway [100]
$t_{39}$	cardiac contractile dysfunction [49]	$t_{88}$	the FAS-FASL interaction [99]
$t_{40}$	cardio-vascular disease symptoms [49]	$t_{89}$	the proteasome pathway in activated SMC [80]
$t_{41}$	modulation of T helper cells type 1 (Th1) differentiation during infections	$t_{90}$	smooth muscles cells (SMC) activation mechanisms [80]
$t_{42}$	reactive oxygen species (ROS) generation in chronic kidney disease (CKD) especially during hemodialysis (HD) [49]	$t_{91}$	IFNγ secretion
$t_{43}$	cardiovascular events [49]	$t_{92}$	regulation of migration and infiltration of monocytes/macrophages
$t_{44}$	BH${}_{3}$ interacting-domain death agonist (BID) cleavage influenced by high levels of active caspase 8 in type I cells [94]	$t_{93}$	mediation of the adhesion of lymphocytes, monocytes, eosinophils, and basophils to vascular endothelium [101]
$t_{45}$	kinase (IKK) complex activation [80]	$t_{94}$	mediation of the adhesion between integrins on leukocytes and endothelial or epithelial cells [101]
$t_{46}$	translocation of truncated BH${}_{3}$ interacting-domain death agonist (BID) to mitochondria [92,94]	$t_{95}$	an inflammatory environment [36]
$t_{47}$	release of cytochrome c from mitochondria to cytosol via mitochondrial perturbation [94]	$t_{96}$	TRAIL production by different cells
$t_{48}$	activation of caspase 9 [94]	$t_{97}$	IL-18 synthesis mediated by caspase 1-independent pathway

## Appendix C. List of t-Clusters

**Table A3:** List of t-clusters. The column “t-clusters” includes the names of t-clusters, the column “t-invariants” includes the names of t-invariants which are included in the particular t-cluster, the column “MCT set” includes the names of MCT set which are related to particular t-invariant and in the column “single transitions” two transitions have been distinguished: $t_{3}$—activation of caspase 8 (marked with blue color) and $t_{97}$—IL-18 synthesis mediated by caspase 1-independent pathway (marked with green color).

t-Cluster	t-Invariants	MCT Set	Single Transitions
$c_{1}$	$x_{1}$	$m_{16}$, $m_{19}$, $m_{20}$	$t_{3}$, $t_{50}$
$c_{2}$	$x_{2}$	$m_{2}$, $m_{14}$, $m_{15}$, $m_{16}$, $m_{20}$	$t_{3}$, $t_{84}$
$c_{3}$	$x_{3}$	$m_{14}$, $m_{15}$, $m_{16}$, $m_{17}$, $m_{20}$	$t_{3}$, $t_{4}$, $t_{84}$
$c_{4}$	$x_{4}$	$m_{15}$, $m_{17}$	$t_{10}$, $t_{34}$, $t_{84}$, $t_{92}$, $t_{97}$
$c_{5}$	$x_{5}$	$m_{4}$, $m_{6}$, $m_{9}$, $m_{11}$, $m_{14}$, $m_{15}$, $m_{16}$, $m_{18}$, $m_{20}$	$t_{3}$, $t_{4}$, $t_{6}$, $t_{10}$, $t_{11}$, $t_{50}$, $t_{84}$
	$x_{6}$	$m_{1}$, $m_{4}$, $m_{6}$, $m_{8}$, $m_{14}$, $m_{15}$, $m_{16}$, $m_{18}$, $m_{20}$	$t_{3}$, $t_{4}$, $t_{6}$, $t_{10}$, $t_{11}$, $t_{24}$, $t_{32}$, $t_{84}$
	$x_{10}$	$m_{1}$, $m_{3}$, $m_{4}$, $m_{5}$, $m_{6}$, $m_{9}$, $m_{11}$, $m_{14}$, $m_{15}$, $m_{16}$, $m_{18}$, $m_{20}$, $m_{21}$	$t_{3}$, $t_{4}$, $t_{6}$, $t_{10}$, $t_{11}$, $t_{84}$, $t_{97}$
	$x_{26}$	$m_{1}$, $m_{4}$, $m_{6}$, $m_{9}$, $m_{11}$, $m_{14}$, $m_{15}$, $m_{16}$, $m_{18}$, $m_{20}$, $m_{21}$	$t_{3}$, $t_{4}$, $t_{6}$, $t_{10}$, $t_{11}$, $t_{24}$, $t_{63}$, $t_{66}$, $t_{84}$, $t_{97}$
	$x_{28}$	$m_{1}$, $m_{4}$, $m_{6}$, $m_{14}$, $m_{15}$, $m_{16}$, $m_{18}$, $m_{19}$, $m_{20}$, $m_{21}$	$t_{3}$, $t_{4}$, $t_{6}$, $t_{10}$, $t_{11}$, $t_{24}$, $t_{63}$, $t_{66}$, $t_{84}$, $t_{97}$
	$x_{31}$	$m_{1}$, $m_{4}$, $m_{6}$, $m_{9}$, $m_{11}$, $m_{14}$, $m_{15}$, $m_{16}$, $m_{18}$, $m_{20}$, $m_{21}$	$t_{3}$, $t_{4}$, $t_{6}$, $t_{10}$, $t_{11}$, $t_{24}$, $t_{63}$, $t_{68}$, $t_{84}$, $t_{97}$
	$x_{33}$	$m_{1}$, $m_{4}$, $m_{6}$, $m_{14}$, $m_{15}$, $m_{16}$, $m_{18}$, $m_{19}$, $m_{20}$, $m_{21}$	$t_{3}$, $t_{4}$, $t_{6}$, $t_{10}$, $t_{11}$, $t_{24}$, $t_{63}$, $t_{68}$, $t_{84}$, $t_{97}$
	$x_{45}$	$m_{1}$, $m_{4}$, $m_{6}$, $m_{8}$, $m_{9}$, $m_{11}$, $m_{12}$, $m_{14}$, $m_{15}$, $m_{16}$, $m_{18}$, $m_{20}$, $m_{21}$	$t_{3}$, $t_{4}$, $t_{6}$, $t_{10}$, $t_{11}$, $t_{24}$, $t_{84}$, $t_{91}$
	$x_{46}$	$m_{1}$, $m_{4}$, $m_{6}$, $m_{8}$, $m_{12}$, $m_{14}$, $m_{15}$, $m_{16}$, $m_{18}$, $m_{19}$, $m_{20}$, $m_{21}$	$t_{3}$, $t_{4}$, $t_{6}$, $t_{10}$, $t_{11}$, $t_{24}$, $t_{84}$, $t_{91}$
	$x_{48}$	$m_{1}$, $m_{4}$, $m_{6}$, $m_{14}$, $m_{15}$, $m_{16}$, $m_{19}$, $m_{20}$, $m_{21}$, $m_{22}$	$t_{3}$, $t_{4}$, $t_{6}$, $t_{10}$, $t_{11}$, $t_{24}$, $t_{84}$, $t_{91}$, $t_{92}$, $t_{97}$
	$x_{50}$	$m_{1}$, $m_{4}$, $m_{6}$, $m_{7}$, $m_{9}$, $m_{11}$, $m_{14}$, $m_{15}$, $m_{16}$, $m_{18}$, $m_{20}$, $m_{21}$	$t_{3}$, $t_{4}$, $t_{6}$, $t_{10}$, $t_{11}$, $t_{24}$, $t_{84}$, $t_{91}$, $t_{97}$
	$x_{52}$	$m_{1}$, $m_{4}$, $m_{6}$, $m_{7}$, $m_{14}$, $m_{15}$, $m_{16}$, $m_{18}$, $m_{19}$, $m_{20}$, $m_{21}$	$t_{3}$, $t_{4}$, $t_{6}$, $t_{10}$, $t_{11}$, $t_{24}$, $t_{84}$, $t_{91}$, $t_{97}$
	$x_{55}$	$m_{1}$, $m_{4}$, $m_{5}$, $m_{6}$, $m_{9}$, $m_{11}$, $m_{14}$, $m_{15}$, $m_{16}$, $m_{18}$, $m_{20}$, $m_{21}$	$t_{3}$, $t_{4}$, $t_{6}$, $t_{10}$, $t_{11}$, $t_{24}$, $t_{84}$, $t_{85}$, $t_{91}$, $t_{97}$
	$x_{57}$	$m_{1}$, $m_{4}$, $m_{5}$, $m_{6}$, $m_{14}$, $m_{15}$, $m_{16}$, $m_{18}$, $m_{19}$, $m_{20}$, $m_{21}$	$t_{3}$, $t_{4}$, $t_{6}$, $t_{10}$, $t_{11}$, $t_{24}$, $t_{84}$, $t_{85}$, $t_{91}$, $t_{97}$
	$x_{63}$	$m_{1}$, $m_{4}$, $m_{6}$, $m_{9}$, $m_{11}$, $m_{14}$, $m_{15}$, $m_{16}$, $m_{18}$, $m_{20}$, $m_{21}$	$t_{3}$, $t_{4}$, $t_{6}$, $t_{10}$, $t_{11}$, $t_{24}$, $t_{31}$, $t_{84}$, $t_{91}$, $t_{97}$
	$x_{66}$	$m_{1}$, $m_{4}$, $m_{6}$, $m_{9}$, $m_{11}$, $m_{14}$, $m_{15}$, $m_{16}$, $m_{18}$, $m_{20}$, $m_{21}$, $m_{22}$	$t_{3}$, $t_{4}$, $t_{6}$, $t_{10}$, $t_{11}$, $t_{24}$, $t_{84}$, $t_{91}$, $t_{92}$, $t_{97}$
$c_{6}$	$x_{7}$	$m_{4}$, $m_{6}$, $m_{9}$, $m_{11}$, $m_{16}$, $m_{18}$, $m_{20}$	$t_{6}$, $t_{10}$, $t_{11}$, $t_{34}$, $t_{50}$, $t_{92}$, $t_{97}$
$c_{7}$	$x_{8}$	$m_{1}$, $m_{2}$, $m_{3}$, $m_{4}$, $m_{5}$, $m_{6}$, $m_{9}$, $m_{11}$, $m_{14}$, $m_{15}$, $m_{16}$, $m_{18}$, $m_{20}$, $m_{21}$	$t_{3}$, $t_{6}$, $t_{10}$, $t_{11}$, $t_{34}$, $t_{92}$, $t_{97}$
	$x_{11}$	$m_{1}$, $m_{3}$, $m_{4}$, $m_{5}$, $m_{6}$, $m_{9}$, $m_{11}$, $m_{14}$, $m_{15}$, $m_{16}$, $m_{18}$, $m_{20}$, $m_{21}$	$t_{3}$, $t_{4}$, $t_{6}$, $t_{10}$, $t_{11}$, $t_{34}$, $t_{92}$, $t_{97}$
	$x_{12}$	$m_{1}$, $m_{3}$, $m_{4}$, $m_{5}$, $m_{6}$, $m_{9}$, $m_{11}$, $m_{15}$, $m_{17}$, $m_{18}$, $m_{21}$	$t_{6}$, $t_{10}$, $t_{11}$, $t_{34}$, $t_{92}$, $t_{97}$
	$x_{17}$	$m_{1}$, $m_{4}$, $m_{6}$, $m_{8}$, $m_{18}$	$t_{6}$, $t_{10}$, $t_{11}$, $t_{24}$, $t_{32}$, $t_{34}$, $t_{92}$, $t_{97}$
	$x_{20}$	$m_{1}$, $m_{4}$, $m_{6}$, $m_{9}$, $m_{10}$, $m_{16}$, $m_{18}$, $m_{19}$	$t_{3}$, $t_{6}$, $t_{10}$, $t_{11}$, $t_{24}$, $t_{34}$, $t_{50}$, $t_{92}$, $t_{97}$
	$x_{22}$	$m_{1}$, $m_{4}$, $m_{6}$, $m_{9}$, $m_{10}$, $m_{11}$, $m_{16}$, $m_{18}$	$t_{6}$, $t_{10}$, $t_{11}$, $t_{24}$, $t_{34}$, $t_{50}$, $t_{92}$, $t_{97}$
	$x_{27}$	$m_{1}$, $m_{4}$, $m_{6}$, $m_{9}$, $m_{11}$, $m_{18}$, $m_{21}$	$t_{6}$, $t_{10}$, $t_{11}$, $t_{24}$, $t_{34}$, $t_{63}$, $t_{66}$, $t_{92}$, $t_{97}$
	$x_{30}$	$m_{1}$, $m_{4}$, $m_{6}$, $m_{9}$, $m_{10}$, $m_{16}$, $m_{18}$, $m_{19}$, $m_{21}$	$t_{3}$, $t_{6}$, $t_{10}$, $t_{11}$, $t_{24}$, $t_{34}$, $t_{63}$, $t_{66}$, $t_{92}$, $t_{97}$
	$x_{32}$	$m_{1}$, $m_{4}$, $m_{6}$, $m_{9}$, $m_{11}$, $m_{18}$, $m_{21}$	$t_{6}$, $t_{10}$, $t_{11}$, $t_{24}$, $t_{34}$, $t_{63}$, $t_{68}$, $t_{92}$, $t_{97}$
	$x_{35}$	$m_{1}$, $m_{4}$, $m_{6}$, $m_{9}$, $m_{10}$, $m_{16}$, $m_{18}$, $m_{19}$, $m_{21}$	$t_{3}$, $t_{6}$, $t_{10}$, $t_{11}$, $t_{24}$, $t_{34}$, $t_{63}$, $t_{68}$, $t_{92}$, $t_{97}$
	$x_{38}$	$m_{1}$, $m_{3}$, $m_{4}$, $m_{5}$, $m_{6}$, $m_{9}$, $m_{10}$, $m_{11}$, $m_{14}$, $m_{15}$, $m_{16}$, $m_{18}$, $m_{21}$	$t_{3}$, $t_{4}$, $t_{6}$, $t_{10}$, $t_{11}$, $t_{24}$, $t_{34}$, $t_{92}$, $t_{97}$
	$x_{39}$	$m_{1}$, $m_{3}$, $m_{4}$, $m_{5}$, $m_{6}$, $m_{9}$, $m_{10}$, $m_{14}$, $m_{15}$, $m_{16}$, $m_{18}$, $m_{19}$, $m_{21}$	$t_{3}$, $t_{4}$, $t_{6}$, $t_{10}$, $t_{11}$, $t_{24}$, $t_{34}$, $t_{92}$, $t_{97}$
	$x_{40}$	$m_{1}$, $m_{2}$, $m_{3}$, $m_{4}$, $m_{5}$, $m_{6}$, $m_{9}$, $m_{10}$, $m_{11}$, $m_{14}$, $m_{15}$, $m_{16}$, $m_{18}$, $m_{21}$	$t_{3}$, $t_{6}$, $t_{10}$, $t_{11}$, $t_{24}$, $t_{34}$, $t_{92}$, $t_{97}$
	$x_{41}$	$m_{1}$, $m_{3}$, $m_{4}$, $m_{5}$, $m_{6}$, $m_{9}$, $m_{10}$, $m_{15}$, $m_{16}$, $m_{17}$, $m_{18}$, $m_{19}$, $m_{21}$	$t_{3}$, $t_{6}$, $t_{10}$, $t_{11}$, $t_{24}$, $t_{34}$, $t_{92}$, $t_{97}$
	$x_{42}$	$m_{1}$, $m_{2}$, $m_{3}$, $m_{4}$, $m_{5}$, $m_{6}$, $m_{9}$, $m_{10}$, $m_{14}$, $m_{15}$, $m_{16}$, $m_{18}$, $m_{19}$, $m_{21}$	$t_{3}$, $t_{6}$, $t_{10}$, $t_{11}$, $t_{24}$, $t_{34}$, $t_{92}$, $t_{97}$
	$x_{51}$	$m_{1}$, $m_{4}$, $m_{6}$, $m_{7}$, $m_{9}$, $m_{11}$, $m_{18}$, $m_{21}$	$t_{6}$, $t_{10}$, $t_{11}$, $t_{24}$, $t_{34}$, $t_{91}$, $t_{92}$, $t_{97}$
	$x_{54}$	$m_{1}$, $m_{4}$, $m_{6}$, $m_{7}$, $m_{9}$, $m_{10}$, $m_{16}$, $m_{18}$, $m_{19}$, $m_{21}$	$t_{3}$, $t_{6}$, $t_{10}$, $t_{11}$, $t_{24}$, $t_{34}$, $t_{91}$, $t_{92}$, $t_{97}$
	$x_{56}$	$m_{1}$, $m_{4}$, $m_{5}$, $m_{6}$, $m_{9}$, $m_{11}$, $m_{18}$, $m_{21}$	$t_{6}$, $t_{10}$, $t_{11}$, $t_{24}$, $t_{34}$, $t_{85}$, $t_{91}$, $t_{92}$, $t_{97}$
	$x_{59}$	$m_{1}$, $m_{4}$, $m_{5}$, $m_{6}$, $m_{9}$, $m_{10}$, $m_{16}$, $m_{18}$, $m_{19}$, $m_{21}$	$t_{3}$, $t_{6}$, $t_{10}$, $t_{11}$, $t_{24}$, $t_{34}$, $t_{85}$, $t_{91}$, $t_{92}$, $t_{97}$
	$x_{60}$	$m_{1}$, $m_{4}$, $m_{6}$, $m_{8}$, $m_{9}$, $m_{11}$, $m_{12}$, $m_{18}$, $m_{21}$	$t_{6}$, $t_{10}$, $t_{11}$, $t_{24}$, $t_{34}$, $t_{91}$, $t_{92}$, $t_{97}$
	$x_{61}$	$m_{1}$, $m_{4}$, $m_{6}$, $m_{8}$, $m_{12}$, $m_{16}$, $m_{18}$, $m_{19}$, $m_{20}$, $m_{21}$	$t_{3}$, $t_{6}$, $t_{10}$, $t_{11}$, $t_{24}$, $t_{34}$, $t_{91}$, $t_{92}$, $t_{97}$
	$x_{62}$	$m_{1}$, $m_{4}$, $m_{6}$, $m_{8}$, $m_{9}$, $m_{10}$, $m_{12}$, $m_{16}$, $m_{18}$, $m_{19}$, $m_{21}$	$t_{3}$, $t_{6}$, $t_{10}$, $t_{11}$, $t_{24}$, $t_{34}$, $t_{91}$, $t_{92}$, $t_{97}$
	$x_{64}$	$m_{1}$, $m_{4}$, $m_{6}$, $m_{9}$, $m_{11}$, $m_{18}$, $m_{21}$	$t_{6}$, $t_{10}$, $t_{11}$, $t_{24}$, $t_{31}$, $t_{34}$, $t_{91}$, $t_{92}$, $t_{97}$
	$x_{65}$	$m_{1}$, $m_{4}$, $m_{6}$, $m_{9}$, $m_{10}$, $m_{16}$, $m_{18}$, $m_{19}$, $m_{21}$	$t_{3}$, $t_{6}$, $t_{10}$, $t_{11}$, $t_{24}$, $t_{31}$, $t_{34}$, $t_{91}$, $t_{92}$, $t_{97}$
	$x_{67}$	$m_{1}$, $m_{4}$, $m_{6}$, $m_{9}$, $m_{11}$, $m_{18}$, $m_{21}$, $m_{22}$	$t_{6}$, $t_{10}$, $t_{11}$, $t_{24}$, $t_{34}$, $t_{91}$, $t_{92}$, $t_{97}$
	$x_{68}$	$m_{1}$, $m_{4}$, $m_{6}$, $m_{9}$, $m_{10}$, $m_{16}$, $m_{18}$, $m_{19}$, $m_{21}$, $m_{22}$	$t_{3}$, $t_{6}$, $t_{10}$, $t_{11}$, $t_{24}$, $t_{34}$, $t_{91}$, $t_{92}$, $t_{97}$
$c_{8}$	$x_{9}$	$m_{1}$, $m_{2}$, $m_{3}$, $m_{4}$, $m_{5}$, $m_{6}$, $m_{14}$, $m_{15}$, $m_{16}$, $m_{18}$, $m_{19}$, $m_{20}$, $m_{21}$	$t_{3}$, $t_{6}$, $t_{10}$, $t_{11}$, $t_{34}$, $t_{92}$, $t_{97}$
	$x_{13}$	$m_{1}$, $m_{3}$, $m_{4}$, $m_{5}$, $m_{6}$, $m_{14}$, $m_{15}$, $m_{16}$, $m_{18}$, $m_{19}$, $m_{20}$, $m_{21}$	$t_{3}$, $t_{4}$, $t_{6}$, $t_{10}$, $t_{11}$, $t_{84}$, $t_{97}$
	$x_{14}$	$m_{1}$, $m_{3}$, $m_{4}$, $m_{5}$, $m_{6}$, $m_{14}$, $m_{15}$, $m_{16}$, $m_{18}$, $m_{19}$, $m_{20}$, $m_{21}$	$t_{3}$, $t_{4}$, $t_{6}$, $t_{10}$, $t_{11}$, $t_{34}$, $t_{92}$, $t_{97}$
	$x_{15}$	$m_{1}$, $m_{3}$, $m_{4}$, $m_{5}$, $m_{6}$, $m_{15}$, $m_{16}$, $m_{17}$, $m_{18}$, $m_{19}$, $m_{20}$, $m_{21}$	$t_{3}$, $t_{6}$, $t_{10}$, $t_{11}$, $t_{34}$, $t_{92}$, $t_{97}$
$c_{9}$	$x_{16}$	$m_{1}$, $m_{4}$, $m_{6}$, $m_{9}$, $m_{10}$, $m_{14}$, $m_{15}$, $m_{16}$, $m_{18}$	$t_{3}$, $t_{4}$, $t_{6}$, $t_{10}$, $t_{11}$, $t_{24}$, $t_{84}$, $t_{97}$
	$x_{21}$	$m_{1}$, $m_{2}$, $m_{4}$, $m_{6}$, $m_{9}$, $m_{10}$, $m_{14}$, $m_{15}$, $m_{16}$, $m_{18}$	$t_{3}$, $t_{6}$, $t_{10}$, $t_{11}$, $t_{24}$, $t_{34}$, $t_{84}$, $t_{92}$, $t_{97}$
$c_{10}$	$x_{18}$	$m_{1}$, $m_{4}$, $m_{13}$, $m_{18}$	$t_{6}$, $t_{10}$, $t_{11}$, $t_{24}$, $t_{31}$, $t_{32}$, $t_{97}$
	$x_{23}$	$m_{1}$, $m_{4}$, $m_{13}$, $m_{22}$	$t_{6}$, $t_{10}$, $t_{24}$, $t_{32}$, $t_{34}$, $t_{92}$, $t_{97}$
	$x_{25}$	$m_{1}$, $m_{4}$, $m_{7}$, $m_{13}$, $m_{18}$	$t_{6}$, $t_{10}$, $t_{24}$, $t_{32}$, $t_{34}$, $t_{92}$, $t_{97}$
	$x_{37}$	$m_{1}$, $m_{4}$, $m_{5}$, $m_{13}$, $m_{18}$	$t_{6}$, $t_{10}$, $t_{24}$, $t_{32}$, $t_{34}$, $t_{85}$, $t_{92}$, $t_{97}$
	$x_{75}$	$m_{1}$, $m_{4}$, $m_{12}$, $m_{13}$, $m_{18}$	$t_{6}$, $t_{10}$, $t_{24}$, $t_{32}$, $t_{34}$, $t_{63}$, $t_{66}$, $t_{92}$, $t_{97}$
	$x_{76}$	$m_{1}$, $m_{4}$, $m_{12}$, $m_{13}$, $m_{18}$	$t_{6}$, $t_{10}$, $t_{24}$, $t_{32}$, $t_{34}$, $t_{63}$, $t_{68}$, $t_{92}$, $t_{97}$
$c_{11}$	$x_{19}$	$m_{1}$, $m_{4}$, $m_{13}$, $m_{14}$, $m_{15}$, $m_{16}$, $m_{20}$, $m_{22}$	$t_{3}$, $t_{4}$, $t_{6}$, $t_{24}$, $t_{32}$, $t_{84}$, $t_{92}$, $t_{97}$
	$x_{24}$	$m_{1}$, $m_{4}$, $m_{7}$, $m_{13}$, $m_{14}$, $m_{15}$, $m_{16}$, $m_{18}$, $m_{20}$	$t_{3}$, $t_{4}$, $t_{6}$, $t_{24}$, $t_{32}$, $t_{84}$, $t_{97}$
	$x_{36}$	$m_{1}$, $m_{4}$, $m_{5}$, $m_{13}$, $m_{14}$, $m_{15}$, $m_{16}$, $m_{18}$, $m_{20}$	$t_{3}$, $t_{4}$, $t_{6}$, $t_{24}$, $t_{32}$, $t_{84}$, $t_{85}$, $t_{97}$
	$x_{69}$	$m_{1}$, $m_{3}$, $m_{4}$, $m_{5}$, $m_{12}$, $m_{13}$, $m_{14}$, $m_{15}$, $m_{16}$, $m_{18}$, $m_{20}$	$t_{3}$, $t_{4}$, $t_{6}$, $t_{32}$, $t_{84}$, $t_{97}$
	$x_{70}$	$m_{1}$, $m_{4}$, $m_{12}$, $m_{13}$, $m_{14}$, $m_{15}$, $m_{16}$, $m_{18}$, $m_{20}$	$t_{3}$, $t_{4}$, $t_{6}$, $t_{24}$, $t_{32}$, $t_{63}$, $t_{66}$, $t_{84}$, $t_{97}$
	$x_{71}$	$m_{1}$, $m_{4}$, $m_{12}$, $m_{13}$, $m_{14}$, $m_{15}$, $m_{16}$, $m_{18}$, $m_{20}$	$t_{3}$, $t_{4}$, $t_{6}$, $t_{24}$, $t_{32}$, $t_{63}$, $t_{68}$, $t_{84}$, $t_{97}$
$c_{12}$	$x_{29}$	$m_{1}$, $m_{4}$, $m_{6}$, $m_{16}$, $m_{18}$, $m_{19}$, $m_{20}$, $m_{21}$	$t_{3}$, $t_{6}$, $t_{10}$, $t_{11}$, $t_{24}$, $t_{34}$, $t_{63}$, $t_{66}$, $t_{92}$, $t_{97}$
	$x_{34}$	$m_{1}$, $m_{4}$, $m_{6}$, $m_{16}$, $m_{18}$, $m_{19}$, $m_{20}$, $m_{21}$	$t_{3}$, $t_{6}$, $t_{10}$, $t_{11}$, $t_{24}$, $t_{34}$, $t_{63}$, $t_{68}$, $t_{92}$, $t_{97}$
	$x_{47}$	$m_{1}$, $m_{4}$, $m_{6}$, $m_{16}$, $m_{18}$, $m_{19}$, $m_{20}$, $m_{21}$	$t_{3}$, $t_{6}$, $t_{10}$, $t_{11}$, $t_{24}$, $t_{31}$, $t_{91}$, $t_{97}$
	$x_{49}$	$m_{1}$, $m_{4}$, $m_{6}$, $m_{16}$, $m_{19}$, $m_{20}$, $m_{21}$, $m_{22}$	$t_{3}$, $t_{6}$, $t_{10}$, $t_{11}$, $t_{24}$, $t_{34}$, $t_{91}$, $t_{92}$, $t_{97}$
	$x_{53}$	$m_{1}$, $m_{4}$, $m_{6}$, $m_{7}$, $m_{16}$, $m_{18}$, $m_{19}$, $m_{20}$, $m_{21}$	$t_{3}$, $t_{6}$, $t_{10}$, $t_{11}$, $t_{24}$, $t_{34}$, $t_{91}$, $t_{92}$, $t_{97}$
	$x_{58}$	$m_{1}$, $m_{4}$, $m_{5}$, $m_{6}$, $m_{16}$, $m_{18}$, $m_{19}$, $m_{20}$, $m_{21}$	$t_{3}$, $t_{6}$, $t_{10}$, $t_{11}$, $t_{24}$, $t_{34}$, $t_{85}$, $t_{91}$, $t_{92}$, $t_{97}$
$c_{13}$	$x_{43}$	$m_{12}$, $m_{13}$, $m_{14}$, $m_{15}$, $m_{16}$, $m_{20}$	$t_{3}$, $t_{4}$, $t_{6}$, $t_{84}$, $t_{91}$, $t_{97}$
$c_{14}$	$x_{44}$	$m_{12}$, $m_{13}$	$t_{6}$, $t_{10}$, $t_{34}$, $t_{91}$, $t_{92}$, $t_{97}$
$c_{15}$	$x_{72}$	$m_{1}$, $m_{2}$, $m_{3}$, $m_{4}$, $m_{5}$, $m_{12}$, $m_{13}$, $m_{14}$, $m_{15}$, $m_{16}$, $m_{18}$, $m_{20}$	$t_{3}$, $t_{6}$, $t_{10}$, $t_{32}$, $t_{34}$, $t_{92}$, $t_{97}$
	$x_{73}$	$m_{1}$, $m_{3}$, $m_{4}$, $m_{5}$, $m_{12}$, $m_{13}$, $m_{15}$, $m_{17}$, $m_{18}$	$t_{6}$, $t_{10}$, $t_{32}$, $t_{34}$, $t_{92}$, $t_{97}$
	$x_{74}$	$m_{1}$, $m_{3}$, $m_{4}$, $m_{5}$, $m_{12}$, $m_{13}$, $m_{14}$, $m_{15}$, $m_{16}$, $m_{18}$, $m_{20}$	$t_{3}$, $t_{4}$, $t_{6}$, $t_{10}$, $t_{32}$, $t_{34}$, $t_{92}$, $t_{97}$
	$x_{77}$	$m_{1}$, $m_{3}$, $m_{4}$, $m_{5}$, $m_{6}$, $m_{9}$, $m_{10}$, $m_{12}$, $m_{13}$, $m_{14}$, $m_{15}$, $m_{16}$, $m_{18}$	$t_{3}$, $t_{4}$, $t_{6}$, $t_{10}$, $t_{11}$, $t_{24}$, $t_{32}$, $t_{34}$, $t_{92}$, $t_{97}$
	$x_{78}$	$m_{1}$, $m_{2}$, $m_{3}$, $m_{4}$, $m_{5}$, $m_{6}$, $m_{9}$, $m_{10}$, $m_{12}$, $m_{13}$, $m_{14}$, $m_{15}$, $m_{16}$, $m_{18}$	$t_{3}$, $t_{6}$, $t_{10}$, $t_{11}$, $t_{24}$, $t_{32}$, $t_{34}$, $t_{92}$, $t_{97}$
